# Nitric oxide-induced ribosome collision activates ribosomal surveillance mechanisms

**DOI:** 10.1038/s41419-023-05997-5

**Published:** 2023-07-26

**Authors:** Laura Ryder, Frederic Schrøder Arendrup, José Francisco Martínez, Goda Snieckute, Chiara Pecorari, Riyaz Ahmad Shah, Anders H. Lund, Melanie Blasius, Simon Bekker-Jensen

**Affiliations:** 1 Center for Healthy Aging, University of Copenhagen, Blegdamsvej 3B, DK-2200 Copenhagen, Denmark; 2 Center for Gene Expression, Department of Cellular and Molecular Medicine, University of Copenhagen, Blegdamsvej 3B, DK-2200 Copenhagen, Denmark; 3 Biotech Research and Innovation Centre, University of Copenhagen, Ole Maaløes Vej 5, DK-2200 Copenhagen, Denmark; 4 Danish Cancer Society Research Center, Strandboulevarden 49, DK-2100 Copenhagen, Denmark

**Keywords:** Ribosomal proteins, Kinases

## Abstract

Impairment of protein translation can cause stalling and collision of ribosomes and is a signal for the activation of ribosomal surveillance and rescue pathways. Despite clear evidence that ribosome collision occurs stochastically at a cellular and organismal level, physiologically relevant sources of such aberrations are poorly understood. Here we show that a burst of the cellular signaling molecule nitric oxide (NO) reduces translational activity and causes ribosome collision in human cell lines. This is accompanied by activation of the ribotoxic stress response, resulting in ZAKα-mediated activation of p38 and JNK kinases. In addition, NO production is associated with ZNF598-mediated ubiquitination of the ribosomal protein RPS10 and GCN2-mediated activation of the integrated stress response, which are well-described responses to the collision of ribosomes. In sum, our work implicates a novel role of NO as an inducer of ribosome collision and activation of ribosomal surveillance mechanisms in human cells.

## Introduction

Ribosomal and translational integrity is essential for cellular and organismal homeostasis. Due to the continuous nature of protein translation, the process is highly sensitive to environmental perturbations. Translational impairment causes ribosomes to slow down, leading to ribosome stalling and collisions, and ribosomes may serve as cellular stress sensors in this capacity [1].

The stress- and mitogen-activated protein kinases (MAPKs) p38 and JNK are activated upon a range of cellular stressors, including reactive oxygen species (ROS), UV radiation, mechanical perturbation, and inflammatory mediators. Downstream signaling from p38 and JNK impacts inflammatory signaling, stress adaptation, and metabolic regulation, and directly affects cell fate decisions such as terminal cell cycle arrest, cell death, and differentiation [2–5]. MAPKs are activated through a three-tiered signal transduction cascade, in which a stress stimulus induces the activating phosphorylation of a MAP kinase kinase kinase (MAP3K). These phosphorylate and activate MAP kinase kinases (MAP2K) which phosphorylate and activate the MAPKs [3, 4, 6]. MAP3Ks constitute a large and diverse kinase family with at least 21 human members with non-overlapping activation mechanisms, of which our knowledge is still limited. One MAP3K that has recently risen to prominence is the ZAKα kinase. Through its binding to the ribosome via two C-terminal domains, ZAKα functions as a sensor of translational impairment [7, 8] and is activated upon stalling and/or collisions of ribosomes [9, 10]. Sensing of such structures induces the autophosphorylation and activation of ZAKα through poorly understood mechanisms and subsequent signaling through the downstream MAPKs, p38, and JNK. Collectively, this pathway is known as the ribotoxic stress response (RSR) [11].

Stalled and collided ribosomes constitute signals for additional surveillance and rescue mechanisms. GCN2, one of the four eIF2α kinases, is also activated by these aberrations to induce integrated stress response (ISR) signaling that enforces transcription and preferential translation of stress-relieving mRNAs [12, 13]. Collided ribosomes must be resolved to avoid cellular proteotoxicity from the accumulation of incomplete polypeptides [1, 14]. This reaction is mediated by the ribosome-associated quality control (RQC) pathway, in which ubiquitination of the ribosomal proteins RPS10 and RPS20 induces splitting of the ribosomal subunits, decay of the mRNA template, and proteasomal degradation of the nascent polypeptide [15]. The key upstream factor for the ubiquitination of collided ribosomes is the E3 ubiquitin ligase ZNF598, which is recruited to the interface between two collided ribosomes [16, 17]. Similar elaborate mechanisms exist for sensing and resolution of individually stalled ribosomes. These depend on the sensing complexes PELOTA-HBS1 [18] and the recently discovered yeast complex Fap1-Smu2-Fpr1 [19].

Most studies that interrogate responses to collided ribosomes are based on cell lines harboring engineered mRNA templates or treated with small-molecule ribosome inhibitors (e.g., anisomycin, cycloheximide) and ribotoxic enzymes (e.g., Shiga toxin, ricin) with unclear physiological relevance [7, 9, 11, 12, 20]. Thus, we are lacking insight into the nature of physiologically as well as pathologically relevant sources of endogenous ribosomal collision. One potential endogenous source of translational stress is nitric oxide (NO), which has been described to inhibit protein translation across different mammalian cell lines [21]. NO is a gaseous paracrine and autocrine signaling molecule with a variety of functions in the body, including its role as a vasodilator in the cardiovascular system, its function as a retrograde neurotransmitter, and its antimicrobial, cytotoxic, and proinflammatory effects in immunity [22–26]. The impact of NO is highly dependent on its concentration, ranging from low levels (nM) in the cardiovascular system to high, pathological levels (μM) as those produced by macrophages and neutrophils upon infection [22, 24, 27, 28]. NO production is mediated by NO synthases (NOS), which catalyze the production of NO from L-arginine and oxygen [25, 29]. There are three NOS isoforms: the endothelial (eNOS), the neuronal (nNOS), and the inducible (iNOS) [25, 29]. Of these, it is primarily the immunological stimulus-induced expression of iNOS that is linked to the production of high, cytotoxic concentrations of NO [22, 28]. NO is a free radical and a highly reactive molecule. The antimicrobial function of NO is non-target specific, damaging macromolecules such as DNA, lipids, and proteins [23, 28, 30]. Such damage has mainly been linked to the production of reactive nitrogen species (RNS), which are generated when NO reacts with other radicals including reactive oxygen species (ROS) in cells [27, 30].

A few studies have linked NO-induced stress to the activation of p38 [31–34] and JNK [34–36] in mammalian cells, however, the physiological mechanisms and relevance of these responses are unclear. Moreover, NO has been shown to both suppress translation and activate the ISR [21]. Here we show that high levels of NO inhibit translation in vivo and in vitro. NO causes ribosome collision and activation of the RSR, the ISR, and the RQC, the three major systems that respond to these structures. Our results indicate that NO, at least at high concentrations, is a physiologically relevant inducer of ribosome collisions and ribosomal surveillance pathways.

## Results

### Nitric oxide induction in cells leads to ZAK-dependent activation of p38 and JNK

**Fig. 1:**
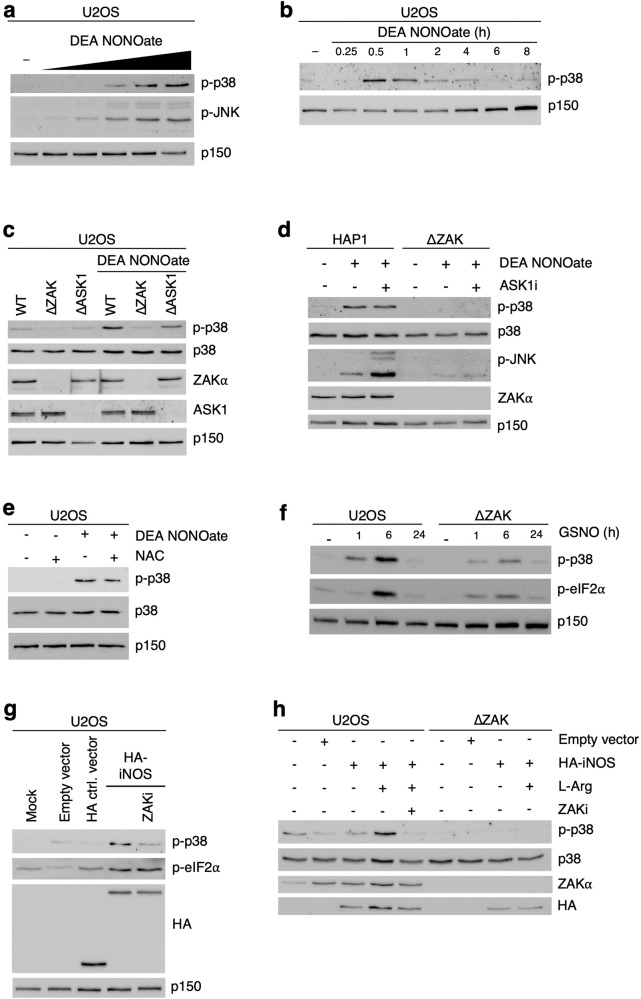
Nitric oxide activates p38 and JNK in a ZAK-dependent manner. **a** U2OS cells were treated with increasing concentrations of DEA NONOate (100 μM, 250 μM, 500 μM, 750 μM, 1000 μM – 1 h). Lysates were analyzed by immunoblotting with the indicated antibodies. **b** U2OS cells were treated with DEA NONOate (750 μM) for the indicated times. Lysates were analyzed as in (**a**). **c** U2OS cells deleted for ZAK (ΔZAK) or ASK1 (ΔASK1) were treated with DEA NONOate (750 μM – 1 h). Lysates were analyzed as in (**a**). **d** HAP1 WT and ΔZAK cells were pretreated with ASK1 inhibitor (2 μM – 30 min) prior to treatment with DEA NONOate (750 μM – 1 h). Lysates were analyzed as in (**a**). **e** U2OS cells were pretreated with N-acetyl-L-cysteine (NAC, 10 mM – 1 h) followed by DEA NONOate treatment (750 μM – 1 h). Lysates were analyzed as in (**a**). **f** U2OS WT and ΔZAK cells were treated with S-Nitrosoglutathione (GSNO, 2 mM) for the indicated times. Lysates were analyzed as in (**a**). **g** U2OS cells were transfected either with empty vector or strep-HA-iNOS and treated with ZAK inhibitor (2 μM – 1 h). Lysates were analyzed as in (**a**). **h** U2OS WT and ΔZAK cells were transfected with empty vector or strep-HA-iNOS and supplemented with L-arginine (10 mM – 20 h) and ZAK inhibitor (2 μM – 1 h). Lysates were analyzed as in (**a**).

By treating U2OS cells with the synthetic, short-lived NO donor DEA NONOate [37], we confirmed published findings [31–36] that NO activates the stress-activated MAPKs p38, and JNK (Fig. 1a). This response was dose-dependent (Fig. 1a) and peaked at half an hour after DEA NONOate treatment (Figs. 1b and S1a), likely reflecting the rapid decomposition of this donor and the gaseous properties of NO. High cellular levels of NO cause oxidative stress via RNS formation [27], which has been shown to activate the MAP3K ASK1 [38]. However, CRISPR/Cas9-mediated knockout (KO) of the *Ask1* gene in U2OS cells or inhibition or KO of ASK1/*Ask1* in human haploid HAP1 cells did not significantly reduce DEA NONOate-induced p38 and JNK activation (Figs. 1c, d and S1b, c). Instead, KO of the Z*ak* MAP3K gene in both U2OS and HAP1 cells completely abrogated the response (Figs. 1c, d and S1b, c). ROS production did not appear to play an important role in these early events, as pre-treatment of cells with the ROS-scavenger N-acetyl-L-cysteine (NAC) had little protective effect (Fig. 1e). Importantly, these effects were not restricted to DEA NONOate as an NO donor. The physiological NO-carrier S-Nitrosoglutathione (GSNO), which exhibits a much longer half-life (hours) than DEA NONOate (minutes), also transiently activated p38, however with a peak effect only after several hours (Fig. 1f). This response was only partially dependent on ZAK, likely due to a protracted low-level release of NO from GSNO that may simultaneously activate other MAP3Ks such as ROS-responsive ASK1. Indeed, with this NO donor, p38 and JNK activation were abrogated by KO of both *Zak* and *Ask1* in an additive fashion (Fig. S1d). Transient expression of the NO-generating enzyme iNOS also caused ZAK-dependent activation of p38 (Fig. 1g), and this response was exacerbated by the addition of the iNOS substrate L-arginine (Fig. 1h). Our results indicate that high levels of NO, whether released by synthetic NO donors or produced by the iNOS enzyme, lead to activation of p38 and JNK in a ZAK-dependent manner.

### Nitric oxide inhibits protein translation and activates the ribotoxic stress response in vivo

**Fig. 2:**
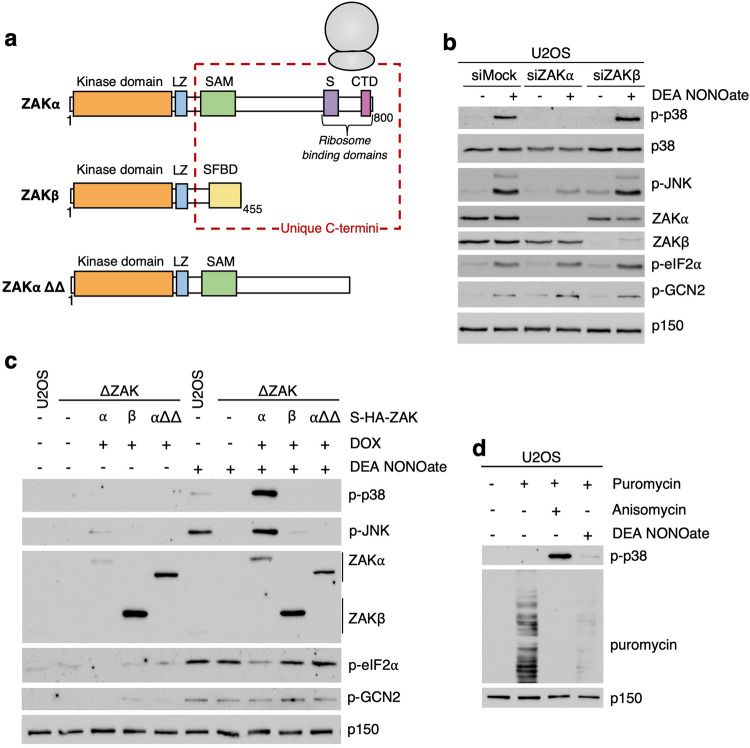
Nitric oxide donors inhibit translation and activate the ribotoxic stress response. **a** Schematic of ZAK protein isoforms and the ZAKα ΔSΔCTD mutant. LZ, Leucine Zipper; SAM, Sterile Alpha-Motif; S, Sensor Domain; CTD, C-Terminal Domain; SFBD, Stress Fiber Binding Domain. **b** U2OS cells were mock-transfected or transfected with siRNAs targeting the α or the β isoform of ZAK. Cells were treated with DEA NONOate (750 μM – 1 h). Lysates were analyzed by immunoblotting with the indicated antibodies. **c** U2OS cells, ΔZAK and ΔZAK cells were rescued with WT ZAKα or ZAKβ, and a mutated form (αΔΔ) of ZAKα were treated with DEA NONOate (750 μM – 1 h). Lysates were analyzed as in (**b**). **d** U2OS cells were treated with DEA NONOate (750 μM – 1 h) or anisomycin (1 μg/ml – 1 h) followed by treatment with puromycin (10 μg/mL - 10 min). Lysates were analyzed as in (b).

The *Zak* gene encodes two alternative splice variants, ZAKα and ZAKβ (Fig. 2a) [6] with unrelated sensing mechanisms. ZAKα is the proximal sensor of ribotoxic stress and translational impairment and interacts directly with the ribosome via a sensor domain (S) and a C-terminal domain (CTD) (Fig. 2a) [7, 9]. ZAKβ senses the deformation of stress fibers and is activated upon volumetric compression of cells and muscle contraction [39]. DEA NONOate-induced activation of p38 and JNK was mediated by ZAKα, but not ZAKβ (Fig. 2b), and required the integrity of the ribosome-binding domains in ZAKα [7] (Fig. 2c). These findings indicate that the responses described above constitute a ribotoxic stress response. Indeed, DEA NONOate strongly impaired the incorporation of the aminoacyl-tRNA analog puromycin into elongating nascent peptides (Fig. 2d). These puromycin-peptide conjugates are prematurely released from the ribosome, and their abundance offers a snapshot of translational activity in cells. Similar effects were observed for GSNO-treated U2OS cells (Fig. S1e), and collectively, these results highlight NO as a potential source of ribotoxic stress.

### Nitric oxide primarily impairs the function of soluble translation factors

**Fig. 3:**
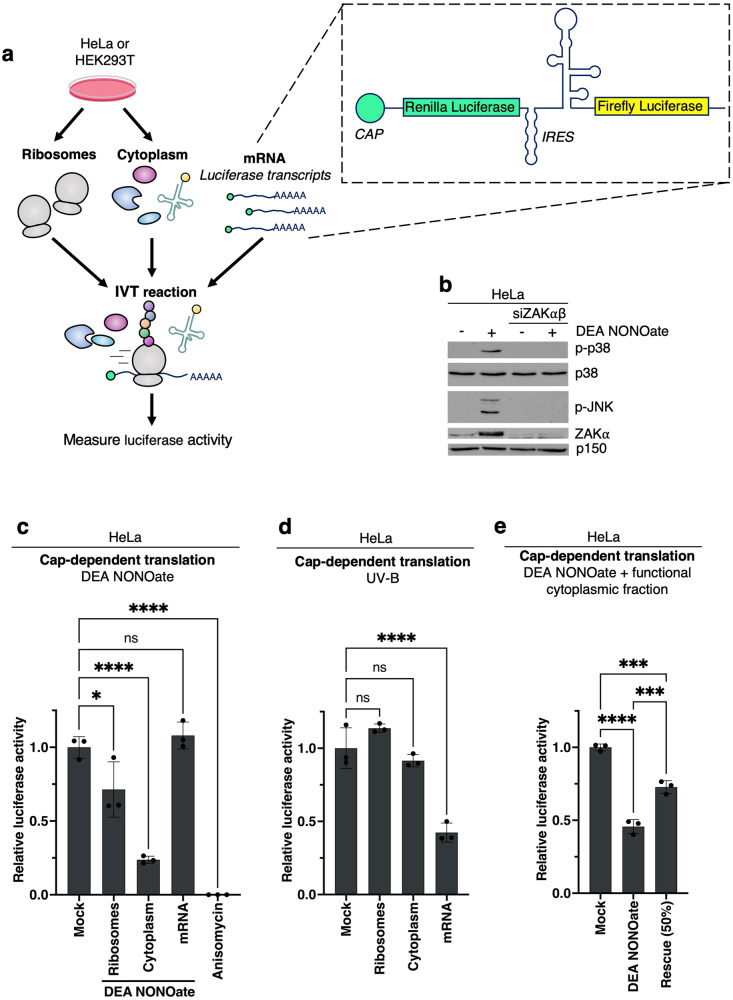
Soluble translation factors are sensitive to nitrosative damage. **a** Schematic of tri-partite in vitro translation (IVT) assay. Ribosomes and a ribosome-depleted cytoplasmic fraction were isolated from cultured HeLa or HEK293 cells and combined with in vitro transcribed luciferase mRNA for IVT. The three fractions can be individually treated before combination. **b** HeLa cells were mock-transfected or transfected with siRNAs targeting both isoforms (α and β) of ZAK. Cells were treated with DEA NONOate (750 μM – 1 h). Lysates were analyzed by immunoblotting with the indicated antibodies. **c** HeLa cell fractions from (**a**) were individually treated with DEA NONOate (1 mM – 10 min) prior to IVT (30 min, 37 °C). Anisomycin (1 μg/ml) was added directly into the full IVT mix as a positive control for translational inhibition. Cap-dependent translation efficiency in the combined reaction was determined by luciferase assay. **d** As in (**c**), except that the fractions were irradiated with UV-B (500 J/m^2^). **e** As in (**c**), except that untreated cytoplasmic fraction was added to the NONOate-treated cytoplasmic fraction before reconstitution of the IVT reaction. Luciferase values were normalized to the mock condition and are plotted as mean. All error bars represent the standard deviation (SD), *n* = 3 technical replicates. ns., non-significant; *p ≤ 0.05; **p ≤ 0.01; ***p ≤ 0.001, ****p ≤ 0.0001 in one-way ANOVA with Dunnett correction for multiple comparisons.

To further investigate the effects of NO on protein synthesis, we set up a tri-partite in vitro translation assay (Fig. 3a). Cellular extracts were split into a ribosomal fraction and a ribosome-depleted cytoplasmic fraction containing tRNAs and initiation and elongation factors. An mRNA allowing for cap-dependent translation of Renilla luciferase and IRES-dependent translation of Firefly luciferase was produced by in vitro transcription. Combination of the three fractions allows for reconstitution of the in vitro translation reaction (Fig. 3a). We isolated the cellular fractions from either HeLa or HEK293 cells, and importantly, DEA NONOate activated p38 and JNK in a ZAK-dependent manner in both cell lines (Figs. 3b, S2a). We treated these three fractions individually with DEA NONOate for 10 min and subsequently combined each of the treated fractions with untreated fractions for in vitro translation. The ability of the cytoplasmic fraction to support the reaction was decreased by approximately 75% for both cap-dependent (Figs. 3c, S2b) and IRES-dependent translation (Fig. S2c, d) when isolated from either cell line. These effects are similar to what we observed when treating intact U2OS cells with DEA NONOate (Fig. 2d). Importantly, the addition of anisomycin to the combined in vitro reaction completely abolished translation and luciferase activity (Figs. 3c, S2b–d). We also observed a minor reduction (approx. 25%) of in vitro translation activity when the ribosomal fraction was treated with DEA NONOate. Conversely, treatment of the mRNA fraction had little to no effect on translational activity (Figs. 3c, S2b–d), suggesting that direct nitrosylation damage to mRNA did not occur to an extent that impacted translational output. This was in contrast to UV-B irradiation, where only nucleotide damage in the mRNA fraction negatively affected in vitro translation (Fig. 3d), consistent with previous results [8]. Addback of untreated cytoplasmic fraction only partially restored cap-dependent luciferase production (Fig. 3e), indicating that the presence of nitrosylated and/or damaged molecules retains dominant negative effects on the translation reaction. Our results indicate that NO mainly targets soluble proteins and/or tRNAs to inhibit translation in an in vitro setting and that nucleotide damage to the mRNA and ribosome only negligibly contributes to the overall effects.

### Nitric oxide induction is associated with ribosome collision and activation of ribosomal surveillance pathways

**Fig. 4:**
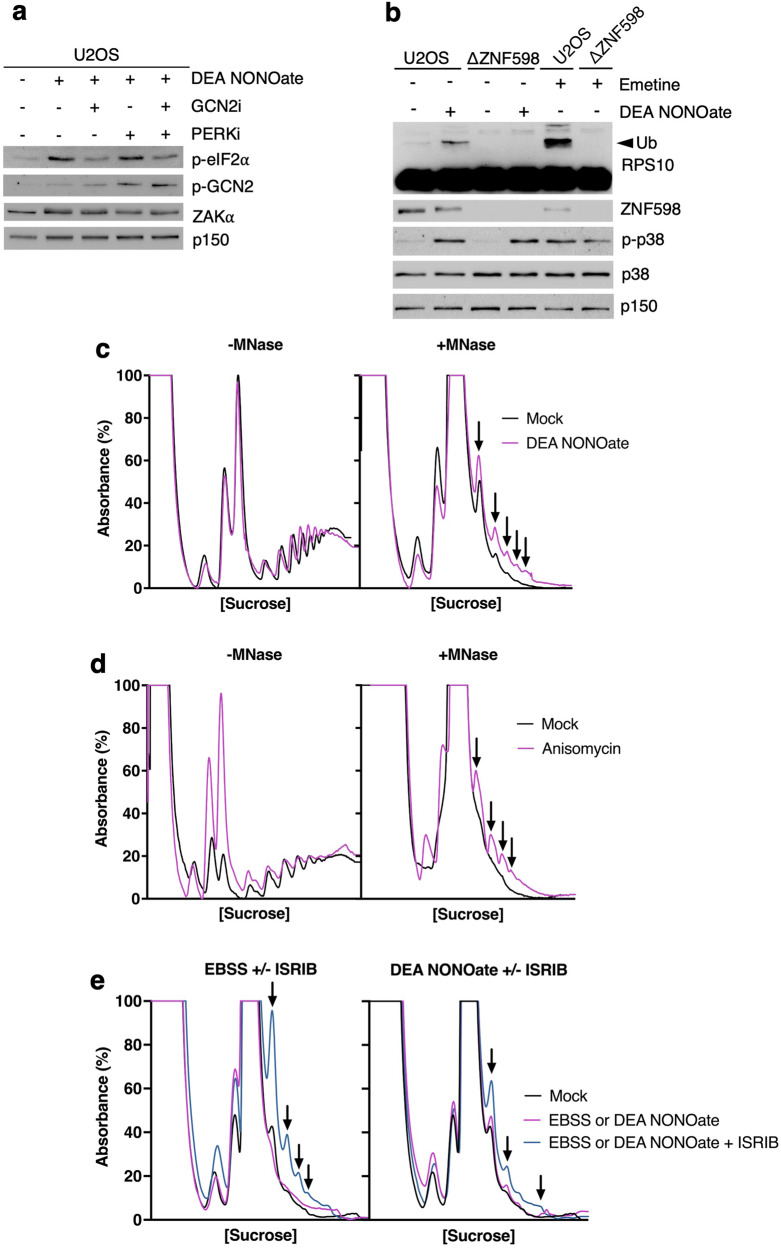
DEA NONOate causes ribosome collision in cells. **a** U2OS cells were pretreated with GCN2 and/or PERK inhibitor (1 μM – 30 min) followed by treatment with DEA NONOate (750 μM – 1 h). Lysates were analyzed by immunoblotting with the indicated antibodies. **b** U2OS WT cells and U2OS cells deleted for ZNF598 (ΔZNF598) were treated with DEA NONOate (750 μM – 1 h). Cells were treated with emetine (1.8 μM – 15 min) as a positive control for RPS10 ubiquitination. Lysates were analyzed as in (**a**). **c** HeLa cells were treated with DEA NONOate (750 μM – 30 min). *Right* – Lysates were digested with micrococcal nuclease (MNase), separated on a linear sucrose gradient, and ribosomes were detected by UV spectrophotometry. *Left* – lysates were not treated with MNase before separation on sucrose gradients. Arrows highlight MNase-resistant di-, tri-, and polysomes, indicative of ribosome collision. **d** As in (**c**), except that cells were treated with anisomycin (1 μg/ml – 15 min). **e** HeLa cells were pre-treated or not with ISRIB (200 nM) and incubated in an EBSS starvation medium (*Left*, 6 h) or DEA NONOate (*Right*, 750 μM – 30 min). Lysates were digested with MNase and analyzed as in (**c**).

The RSR is only one of several translational surveillance pathways activated by ribosome collision [12]. Another one is the ISR, in which cap-dependent translation initiation is blocked via inhibitory phosphorylation of eIF2α [9, 13]. NO release resulted in eIF2α phosphorylation both when cells were treated with DEA NONOate (Fig. 2b, c) and GSNO (Fig. 1f) or when overexpressing iNOS (Fig. 1g). Further, we observed that DEA NONOate induced the phosphorylation of ribosome-associated GCN2, one of the four eIF2α kinases that can activate the ISR (Fig. 2b, c). We also found that inhibition of GCN2 kinase activity impaired DEA NONOate-induced phosphorylation of eIF2α (Fig. 4a). Inhibition of the PKR-like endoplasmic reticulum (ER) kinase (PERK), an eIF2α kinase linked to ER-stress, did not affect eIF2α phosphorylation under these conditions. These data suggest that NO-associated ISR activation is a GCN2-dependent and thus ribosome-templated event.

DEA NONOate treatment also triggered ZNF598-dependent ubiquitination of the small ribosomal protein RPS10 (Figs. 4b, S2e), a proximal event in the RQC pathway and a well-described marker of ribosome collision [16, 17]. To characterize the extent of ribosome collision, we adopted a recently described version of polysome profiling, where in vitro digestion with micrococcal nuclease (MNase) converts polysomes to monosomes [40]. Collided ribosomes protect the associated mRNA from digestion, and appear as peaks corresponding to dismiss, trisomes, etc. when ribosomal material is separated over a sucrose gradient. Using this method, we observed clear signs of collided ribosomes in cells treated with DEA NONOate (Fig. 4c – right). Of note, even though DEA NONOate markedly repressed bulk translation in cells (Fig. 2d), profiles of undigested polysome material looked indistinguishable from mock-treated cells (Fig. 4c – left). The ribosome collision-promoting effects of DEA NONOate were similar to when cells were treated with anisomycin (Fig. 4d), a known inducer of these aberrations. In the presence of stalled ribosomes, ISR signaling prevents new translation initiation events on affected mRNAs. In the absence of this mechanism, the collision of trailing ribosomes with stalled ones ensues [41], exacerbating collision effects. Inhibition of the ISR by the molecule ISRIB [42, 43] elevated NO-induced ribosome collision (Fig. 4e - right), similar to when cells were incubated in an EBSS starvation medium (Fig. 4e - left) as previously shown [10]. Our results highlight NO, at least when released in high amounts, as a novel inducer of ribosome collision that activates all of the known signaling pathways responding to these structures.

## Discussion

**Fig. 5:**
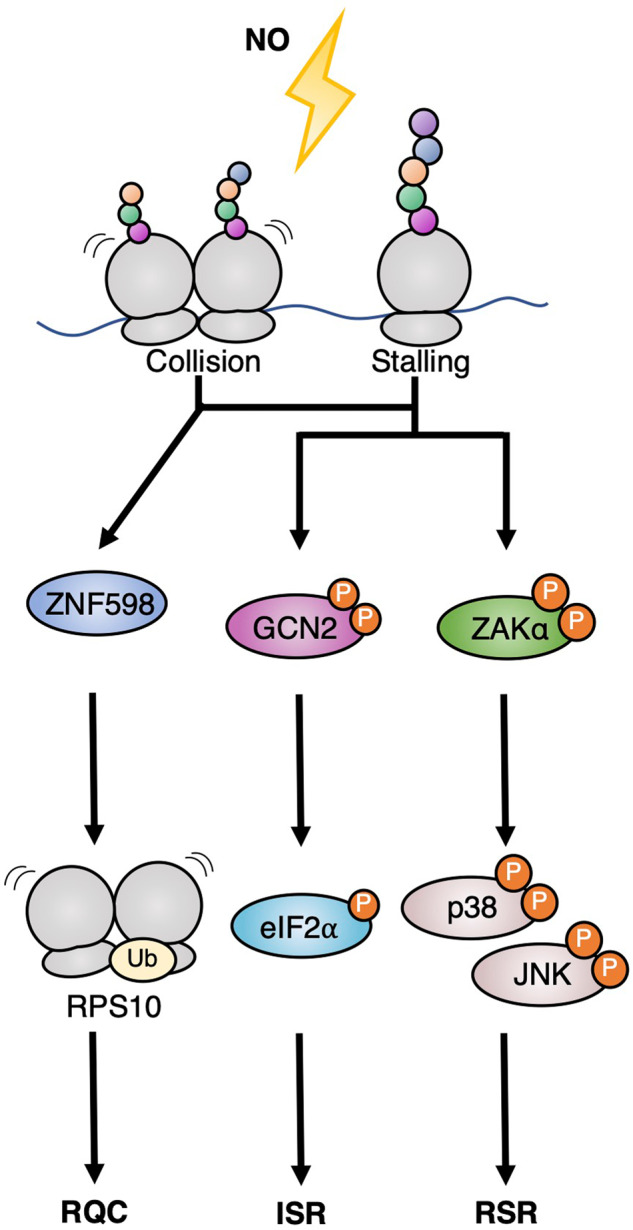
Nitric oxide activates ribosomal surveillance mechanisms. Nitric oxide (NO) induces ribosome collision and stalling to activate the three major collision surveillance systems. RQC ribosome-associated quality control, ISR integrated stress response, RSR ribotoxic stress response.

Here we show that high but physiologically relevant NO levels inhibit translation in vitro and in vivo and induce ZAKα-mediated activation of the p38 and JNK kinases. High NO levels cause ribosome stalling as well as collision (Fig. 4c, e) and activate the three known responses to these structures: the RSR, RQC, and ISR ribosome stress-surveillance pathways (Fig. 5). NO is enzymatically produced in multiple human tissues, has important biological functions, and is produced in high concentrations by immune cells [22–26]. Our findings highlight NO molecules as a physiologically and/or pathologically relevant source of ribotoxic stress and ribosome collision. Given the sparsity of known endogenous sources of such aberrations, this insight is an important early step in shaping our understanding of the biological relevance of ribosomal surveillance pathways in vivo. Previous work along these lines has uncovered UV-B irradiation as an important source of ribosome collision in skin keratinocytes [44, 45], while amino acid deprivation has been shown to mainly cause stalling and/or pausing of individual ribosomes [10]. In both cases, RSR signaling from these structures has been shown to impact physiologically important adaptation responses. Conversely, mice with deficiencies in the RQC pathway succumb to early-onset neurodegeneration, suggesting that especially neuronal cells are challenged by ribosome collision from unknown sources on a stochastic basis [46, 47]. In the case of NO, we do not at present understand the physiological importance of ribosomal stress responses, and this will be an important avenue of future study. One possibility is that cytotoxic NO production achieved by immune cells such as macrophages, neutrophils, and T cells [24, 28] is sufficiently high to cause ribosome collision in the producing cells. In this context, ISR signaling may increase the tolerance of the immune cells that produce NO in response to an acute infection. In addition, the RSR signaling pathway has the potential to enforce immunological signaling and cytokine production in activated and NO-producing immune cells [4]. The tumor microenvironment is another biological niche densely populated with NO-producing cells of both tumor and immune system origin, and NO levels can reach micromolar levels in this milieu [48, 49]. In the context of tumors, NO has both toxic and protective properties [50], and ribosomal surveillance mechanisms such as the RSR, ISR, and RQC pathways are likely to be involved in mediating some of these effects.

Previous studies have shown that NO both suppresses protein translation [21] and induces the activation of the two stress-activated MAPKs, p38 and JNK [31–36]. Our findings highlight that ZAKα, the known MAP3K sensor of ribotoxic stress and transducer of the RSR [7, 9, 11], connects these two phenomena, at least in human cell lines (Fig. 2b, c). ZAKα can be activated both by stalling and collision of ribosomes, with the latter being the more robust activation platform [7, 9, 10]. Treatment of cells with DEA NONOate was accompanied by an increase in MNase-resistant polysome peaks (Fig. 4c, right) and activation of the collision sensor ZNF598 (Fig. 4b; Fig. S2e), which indicate the formation of ribosome collisions. It is entirely possible that only a small part of stalled ribosomes progress to collision, and at present, we do not have insight into the ratio of stalled vs. collided ribosomes. We also corroborated previous findings [21] showing that NO activates the ISR, indicated by the phosphorylation of eIF2α (Fig. 2b, c). Here we report that this reaction is mainly mediated by the ribosome-associated eIF2α kinase GCN2 (Fig. 4a). Similar to ZAKα, but opposite to ZNF598, GCN2 activation can occur in response to several translational problems, including stalling and collision of ribosomes [9, 51–55].

Employing a partitioned in vitro translation assay, we observed that soluble cytoplasmic translation factors, and to a lesser degree ribosomes, were exquisitely sensitive to the effects of NO release from DEA NONOate (Fig. 3c). Conversely, pre-treatment of the mRNA did not impair translation of the template. This suggests that NO, despite being a free radical, does not damage mRNA or rRNA to an extent that blocks translation and that proteins are the target for nitrosylation damage in this context. NO primarily reacts with cysteines in a process known as S-nitrosylation, which can both serve roles in cellular signaling [27, 56, 57] and constitute damaging oxidative modifications. Potential protein targets in the cytoplasmic and ribosomal fractions are initiation and elongation factors as well as ribosomal proteins. Finally, tRNAs in the cytoplasmic fraction may also be relevant targets. Future work is required to elucidate the translation-associated molecular targets of NO modification with the potential to disturb ribosomal function and activate ribosomal surveillance.

In conclusion, our work highlights NO as a reactive molecule with the potential to disturb key cellular processes such as protein translation, induce physiologically relevant ribosomal collision, and evoke ribosomal stress responses.

## Materials and methods

### Cell culture and reagents

Human osteosarcoma cells (U2OS - ATCC), human malignant cervical epithelial cells (HeLa - ATCC), and immortalized human embryonic kidney (HEK293 - ATCC) cells were cultured in Dulbecco’s Modified Eagle’s Medium (DMEM, Biowest) supplemented with 10% fetal bovine serum (FBS, Biowest), L-glutamine, 1% penicillin and streptomycin. Human near haploid cells (HAP1) were cultured in Iscove’s Modified Dulbecco’s Medium (IMDM) GlutaMAX™ Supplement (31980022, Thermo Fisher Scientific) supplemented with 10% FBS, 1% penicillin, and streptomycin. All cells were cultured at 37 °C in a humidified 5% CO_2_ cell incubator. Experiments were conducted when the cells reached 70–80% confluency. Cell lines were not recently authenticated but were tested for mycoplasma contamination on a quarterly basis.

U2OS ZAK KO cells, U2OS ZNF598 KO cells, and HAP1 ZAK KO cells were previously described [7, 38]. Plasmids for CRISPR/Cas9 mediated generation of U2OS ASK1 KO, U2OS ASK1/ZAK KO, HAP1 ASK1 KO, and HAP1 ASK1/ZAK KO cells were cloned using the plasmid pX459 (Addgene #62988). Briefly, gRNA oligos were ordered as complementary sequences, mixed in a 1:1 ratio, and annealed. pX459 was digested with BbsI, and the gRNA was introduced using a standard ligation reaction. The following gRNA sequences were used for ASK1 deletion: ASK1-sgRNA-1: 5′-ATTGTAAAAGCGGTCCAGCACGG and ASK1-sg-RNA-3: 5′-GTGATCAACGAAGCGAGCCAAGG. For U2OS ASK1/ZAK KO cells and HAP1 ASK1/ZAK KO cells, either U2OS ZAK KO or HAP1 ZAK KO cells were transfected with pX459-sgASK1. All constructs were verified by sequencing. Stable cell lines expressing wildtype or truncated versions of ZAKα and ZAKβ were previously described [7].

Chemicals and inhibitors used in this study were: Diethylamine NONOate sodium salt hydrate (DEA NONOate) (Sigma-Aldrich, #D184, 750 μM, 1 h), S-Nitrosoglutathione (GSNO) (Sigma-Aldrich, #N4148, 2 mM, 1–24 h), doxycycline (Sigma-Aldrich, D3347, 0.13 μg/ml, overnight), anisomycin (Sigma-Aldrich, A9789, 1 μg/ml, 1 h), emetine (Sigma-Aldrich, E2375, 1.8 μM, 15 min), puromycin (BioNordika, 13884, 10 μg/ml, 10 min), N-Acetyl-L-cysteine (Sigma-Aldrich, #A7250, 10 mM, 1 h pre-treatment), ZAK inhibitor (2 μM, 30 min pre-treatment, a gift from Xiaoyun Lu (Jinan University, China) [58]), GCN2 inhibitor A-92 (Axon medchem, #2720, 1 μM, 30 min pre-treatment), PERK inhibitor GSK2606414 (SelleckChem, #S7307, 1 μM, 30 min pre-treatment), ASK1 inhibitor Selonsertib, (Selleckchem, #S8292, 2 μM, 30 min pre-treatment) and L-arginine (Sigma-Aldrich, #A8094, 10 mM, 20 h), ISRIB (Sigma-Aldrich, #SML0843-5MG, 200 nM, 30 min pre-treatment), Earle’s Balanced Salts (EBSS) (Sigma-Aldrich, #E3024, 6 h).

### Western blotting and antibodies

For whole cell extracts, cells were lysed in EBC buffer (50 mM Tris, pH 7.5, 150 mM NaCl, 1 mM EDTA, 0.5% NP-40) supplemented with protease and phosphatase inhibitors. Samples were mixed with Laemmli sample buffer and boiled for 5 min. Protein samples were resolved by SDS-PAGE and transferred to nitrocellulose membranes. Membranes were blocked in PBS-Tween + 5% milk before incubation with primary antibody overnight at 4 °C. Next, membranes were washed in PBS-tween and incubated with HRP Goat Anti-Rabbit or Goat Anti-Mouse IgG Antibody (H + L) for 1 h at room temperature. Lastly, membranes were washed in PBS-tween and visualized by chemiluminescence (Clarity Western ECL substrate, Bio-Rad) using the Bio-Rad Chemidoc imaging system. Antibodies used in this study were: anti-phospho-p38 (Cell Signaling, #9216), anti-p38 (Cell Signaling, #9212), anti-phospho-SAPK/JNK (Cell Signaling Technology, #9255), anti-ZAK (Proteintech, #14945-1-AP), anti-ZAKα (Bethyl #A301-993A), anti-p150 (BD biosciences, #610473), anti-phospho-GCN2 (Abcam, #ab75837), anti-phospho-eIF2α (Cell Signaling, #3398), anti-puromycin (Millipore, #MABE343), anti-EDF1 (Abcam, #ab174651), anti-RPS2 (Bethyl, #A303-794A), anti-RPS10 (Abcam, #ab151550), anti-ZNF598 (Abcam, #ab111698), ASK1 (Thermo Scientific, #702278), α-tubulin (Sigma, #T9026) and anti-HA (Santa Cruz, #sc-7392).

### Cloning and transient transfection of iNOS

Full-length iNOS (hNOS2 gene) was subcloned from the pLIX403-hNOS2 plasmid (Addgene plasmid #110800) into a pcDNA4/TO/Strep-HA vector using NotI restriction sites. The following primer sequences were used. hNOS2-Fw: 5′-AAGCGGCCGCGCCTGTCCTTGGAAATTTCTG and hNOS2-Rv: 5′-AAGCGGCCGCTCAGAGCGCTGACATCTCCAG. Transient transfection of U2OS cells with pcDNA4/TO/Strep-HA-hNOS2 was carried out with Polyethylenimine (PEI, 3.1 ng/μl–4.4 ng/μl, 4 h) when cells were 40–50% confluent. Cells were treated and harvested the following day.

### siRNA transfections

Reverse transfection of siRNAs was carried out using Lipofectamine™ RNAiMAX Transfection Reagent (Thermo Fisher Scientific, #13778) following the manufacturer’s instructions. siRNA sequences used in this study were: ZAKα: 5′-GGUGCCCAUUAAGUAUCAA(dTdT), ZAKβ: 5′-CAUGCAAGCCAAGCAGAAU(dTdT) and ZAKαβ: 5′-GCUUAAAGAACGAGAAAGA(dTdT).

### Polysome profiling

After cells were exposed to various treatments, cytosolic lysates were prepared using 20 mM Hepes pH 7.5, 100 mM NaCl, 5 mM MgCl_2_, 100 μg/ml digitonin, 100 μg/ml cycloheximide, 1X protease inhibitor cocktail (Sigma, #P2714) and 200 U RiboLock RNase Inhibitor (Thermo Fisher Scientific, #EO0382). Extracts were passed 10 times through a 26 G needle and incubated on ice for 5 min prior to centrifugation at 17,000 g for 5 min at 4 °C. After adding calcium chloride to a final concentration of 1 mM, lysates were optionally digested with 500 U micrococcal nuclease (MNase) (New England Biolabs, #M0247) for 30 min at 22 °C. Digestion was terminated by adding 2 mM EGTA. Equivalent amounts of lysate (100–120 μg of undigested RNA or 150–180 μg of MNase-digested RNA) were resolved on 15–50% sucrose gradients. Gradients were centrifuged at 38,000 rpm in a Sorvall TH64.1 rotor for 2.5 h at 4 °C. The gradients were passed through a Biocomp density gradient fractionation system with continuous monitoring of the absorbance at 260 nm.

### Tri-partite in vitro translation assay

#### Isolation of ribosomes

For isolation of ribosomes, HeLa or HEK293 cells were cultured to 80% confluency and scraped off in ice-cold PBS. Cells were lysed in polysome lysis buffer (20 mM Tris-HCl, 150 mM KCl, 5 mM MgCl_2_, 0.5% NP40, 2 mM DTT, Roche EDTA-free Protease Inhibitor, and murine RNase inhibitor (New England Biolabs, #M0314)) and incubated while rotating for 10 min at 4 °C. Lysates were cleared by centrifugation at 14,000 g for 10 min and supernatants were recovered and supplemented with 25 µM Hemin (Sigma-Aldrich, #51280). Cleared lysates were loaded on top of a 500 µl 34% sucrose cushion in hypotonic buffer (10 mM HEPES, 10 mM KAc, 0.5 mM MgAc_2_) and centrifuged for 240,000 g, 2 h 15 min, 4 °C. Pellets containing ribosomes were washed thoroughly and resuspended in 0.2 X of loaded lysate volume in hypotonic ribosome buffer (20 mM HEPES, 10 mM NaCl, 25 mM KAc, 1.1 mM MgAc_2_).

#### Isolation of ribosome-free cytoplasm

For isolation of cytoplasm, lysates were prepared as described [59] with some modifications: HeLa or HEK293 cells were cultured to 80% confluency and detached by trypsinization followed by trypsin inactivation. Cells were pelleted by centrifugation at 500 g for 4 min, washed in PBS, and resuspended in equal volumes of hypotonic lysis buffer (10 mM HEPES, 10 mM KAc, 0.5 mM MgAc_2_, 5 mM DTT, Roche EDTA-free Protease Inhibitor) and incubated rotating 1 h, 4 °C. After incubation, lysates were pulled through a 27 G, 3/4-inch needle 8–10 times to lyse the cells, and debris was cleared by centrifugation at 14,000 g for 2 min. Cleared lysates were supplemented with 25 µM Hemin and centrifuged for 240,000 g, 2 h 15 min, 4 °C. Pellets containing ribosomes were discarded and supernatants were recovered as ribosome-free cytoplasmic lysate.

#### Reconstituted in vitro translation

Translation reactions were reconstituted by mixing one-part isolated ribosomes, five-parts cytoplasmic lysates, one-part in vitro transcribed luciferase mRNA (final conc. 1–10 ng/µl), and three-part translation buffer final concentrations of 1.6 mM HEPES, 10 mM creatine phosphate (Sigma Aldrich, #27920), 50 ng/µl creatine kinase (Sigma Aldrich, #10127566001), 10 µM spermidine (Sigma Aldrich, # S0266), 10 µM amino acids (Promega, # L4461), 65 mM KAc, 0.75 mM MgAc_2_ and murine RNase inhibitor A dual mRNA construct with a Renilla luciferase coding sequence under cap-dependent translation control and a Firefly luciferase coding sequence under control of cap-independent EMCV IRES. This construct was transcribed from a HpaI-linarized plasmid using HiScribe™ T7 ARCA mRNA Kit with tailing (New England Biolabs, #E2060) according to the manufacturer’s specifications. Translation reactions were incubated at 37 °C for 30 min and translational output was measured as luciferase activity detected with Dual-Glo® Luciferase Assay System (Promega, # E2940) according to manufacturer’s specifications. For treatment with NO donor, fractions were treated with 1 mM DEA NONOate for 10 min. For UV irradiation, fractions were irradiated with 500 J/m^2^ UV-B and immediately added to the in vitro translation reaction. As a positive control, anisomycin (1 μg/ml) was added to the already combined reaction mix. Based on previous experiences with the system, a sample size of *n* = 3 was chosen for experiments with HeLa cells and *n* = 2 for experiments with HEK293 cells.

### Statistical analysis and data availability

The in vitro translation data was analyzed in Prism 9 using one-way ANOVA either comparing each condition to the mock or comparing the mean of each condition to every other condition with respectively Dunnett correction or Tukey correction for multiple comparisons. A significance level of 0.05 was used and *p*-values were presented as ns., non-significant; *p* > 0.05; *p ≤ 0.05; **p ≤ 0.01; ***p ≤ 0.001; ****p ≤ 0.0001. Results were presented using bar plots of the mean with error bars for the standard deviation (SD). Values from treated samples were normalized to the mean value of the mock. The variance was similar between groups that were statistically compared. Full and uncropped original western blots are available in the "original data file”.

## Supplementary information

Supplemental material

Original Data File

Reproducibility checklist
