# Supplementary material for: Nitric oxide-induced ribosome collision activates ribosomal surveillance mechanisms
Source: Cell Death Dis. 2023 Jul 26;14(7):467. doi: 10.1038/s41419-023-05997-5 (PMC10372077; doi:10.1038/s41419-023-05997-5)
Supplement: Supplementary file 1 — Supplemental material [file 41419_2023_5997_MOESM1_ESM.pdf]

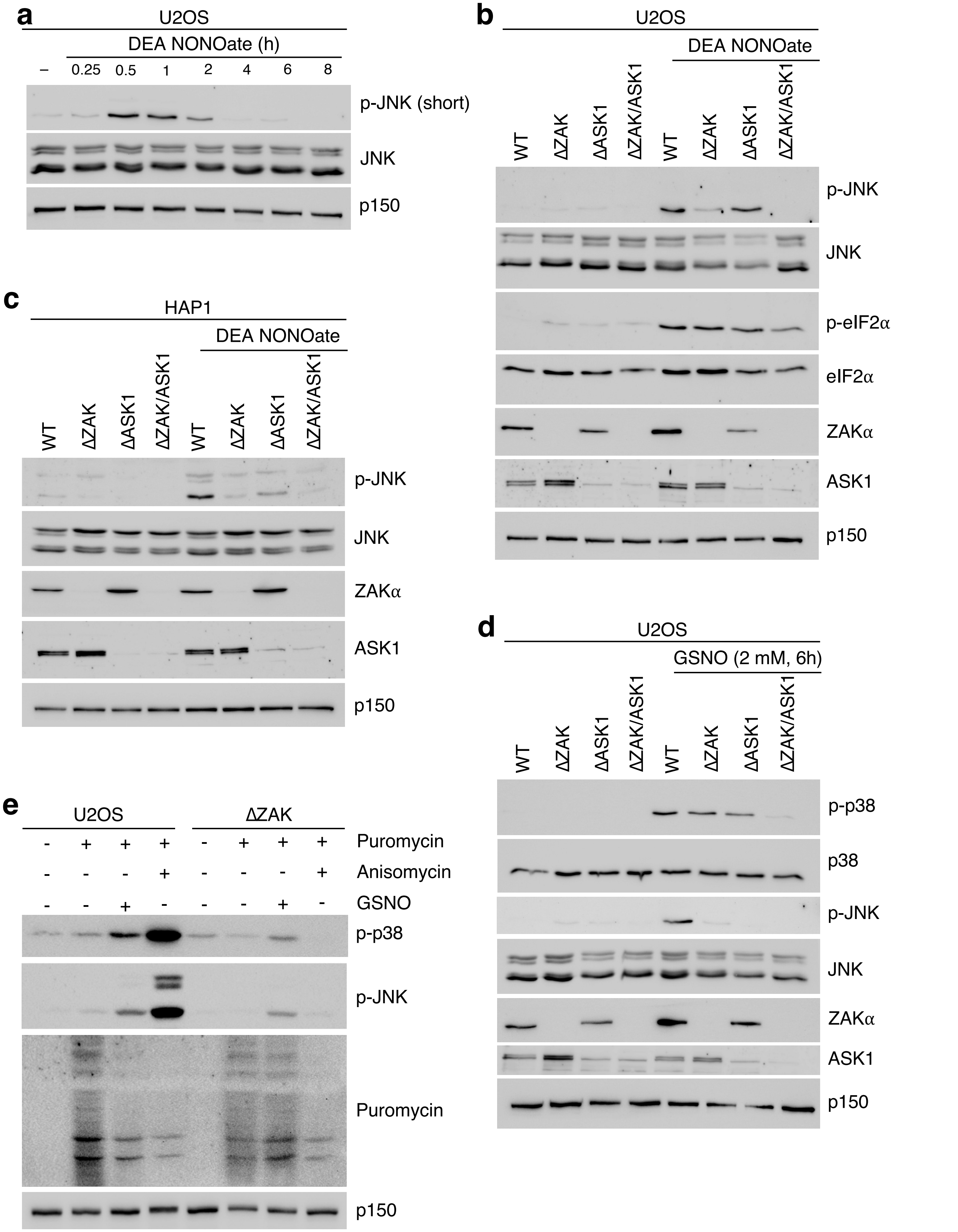
a
U2OS
DEA NONOate (h)
–
0.25
0.5
1
2
4
6
8
p-JNK (short)
JNK
p150
b
U2OS
DEA NONOate
WT
ΔZAK
ΔASK1
ΔZAK/ASK1
p-JNK
JNK
p-eIF2α
eIF2α
ZAKα
ASK1
p150
c
HAP1
DEA NONOate
WT
ΔZAK
ΔASK1
ΔZAK/ASK1
p-JNK
JNK
ZAKα
ASK1
p150
d
U2OS
GSNO (2 mM, 6h)
WT
ΔZAK
ΔASK1
ΔZAK/ASK1
p-p38
p38
p-JNK
JNK
ZAKα
ASK1
p150
e
U2OS
ΔZAK
Puromycin
Anisomycin
GSNO
p-p38
p-JNK
Puromycin
p150

**Figure S1.**

**Involvement of *Zak* and *Ask1* genes in stress responses caused by nitric oxide donors.**

**a.** U2OS cells were treated with DEA NONOate (750 µM) for the indicated times. Lysates were analyzed by immunoblotting with the indicated antibodies. **b.** U2OS WT cells and cells deleted for ZAK (ΔZAK), ASK1 (ΔASK1) and both ZAK and ASK1(ΔZAK/ASK1) were treated with DEA NONOate (750 µM – 1 h). Lysates were analyzed as in (a). **c.** HAP1 WT, ΔZAK, ΔASK1 and ΔZAK/ASK1 cells were treated with DEA NONOate (750 µM – 1 h). Lysates were analyzed as in (a). **d.** Cells from (b) were treated with DEA NONOate (GSNO, 2 mM – 6 h). Lysates were analyzed as in (a). **e.** U2OS WT and ΔZAK cells were treated with S-Nitrosoglutathione (GSNO, 2 mM – 6 h) or anisomycin (1 µg/ml – 1 h) followed by treatment with puromycin (10 µg/mL - 10 min). Lysates were analyzed by immunoblotting with the indicated antibodies.

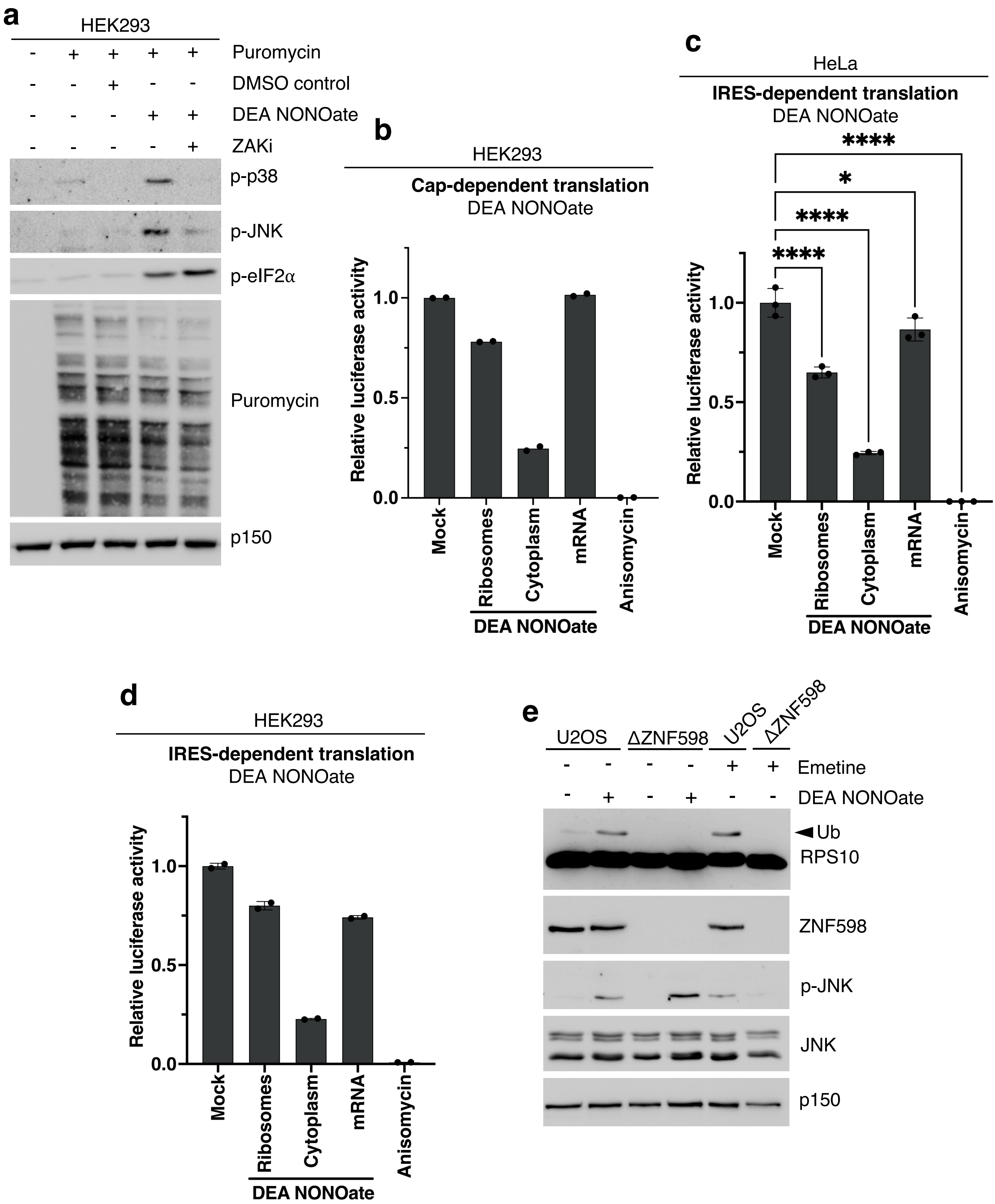
a
HEK293
Puromycin
DMSO control
DEA NONOate
ZAKi
p-p38
p-JNK
p-eIF2α
Puromycin
p150
b
HEK293
Cap-dependent translation
DEA NONOate
Relative luciferase activity
Mock
Ribosomes
Cytoplasm
mRNA
Anisomycin
DEA NONOate
c
HeLa
IRES-dependent translation
DEA NONOate
****
*
****
****
Relative luciferase activity
Mock
Ribosomes
Cytoplasm
mRNA
Anisomycin
DEA NONOate
d
HEK293
IRES-dependent translation
DEA NONOate
Relative luciferase activity
Mock
Ribosomes
Cytoplasm
mRNA
Anisomycin
DEA NONOate
e
U2OS
ΔZNF598
U2OS
ΔZNF598
Emetine
DEA NONOate
Ub
RPS10
ZNF598
p-JNK
JNK
p150

**Figure S2.**

**A tripartite *in vitro* translation system reveals nitric oxide-sensitive translation factors.**

**a.** HEK293 cells were pretreated with ZAK inhibitor (2 µM – 30 min) prior to treatment with DEA NONOate (750 µM – 1 h) or DMSO followed by treatment with puromycin (10 µg/mL - 10 min). Lysates were analyzed by immunoblotting with the indicated antibodies. **b.** HEK293 cell fractions from Figure 3a were individually treated with DEA NONOate (1 mM – 10 min) prior to IVT (30 min, 37 °C). Anisomycin (1 µg/ml) was added directly into the full IVT mix as a positive control for translational inhibition. Cap-dependent translation efficiency in the combined reaction was determined by luciferase assay. n=2 technical replicates. **c.** HeLa cell fractions from Figure 3a were individually treated with DEA NONOate (1 mM – 10 min) prior to IVT (30 min, 37 °C). IRES-dependent translation using HeLa cell fractions was measured by luciferase assay. n=3 technical replicates. **d.** As in (c), except that HEK293 cells were used. n=2 technical replicates. **e.** U2OS WT cells and U2OS cells deleted for ZNF598 (ΔZNF598) were treated with DEA NONOate (750 µM – 1 h). Cells were treated with emetine (1.8 µM – 15 min) as a positive control for RPS10 ubiquitination. Lysates were analyzed as in (a). Luciferase values were normalized to the mock condition and are plotted as mean. All error bars represent the standard deviation (SD). ns., non-significant; *, $p \leq 0.05$; **, $p \leq 0.01$; ***, $p \leq 0.001$, ****, $p \leq 0.0001$ in one-way ANOVA with Dunnett correction for multiple comparisons.
