# Supplementary material for: Nitric oxide-induced ribosome collision activates ribosomal surveillance mechanisms
Source: Cell Death Dis. 2023 Jul 26;14(7):467. doi: 10.1038/s41419-023-05997-5 (PMC10372077; doi:10.1038/s41419-023-05997-5)
Supplement: Supplementary file 2 — Original Data File [file 41419_2023_5997_MOESM2_ESM.pdf]

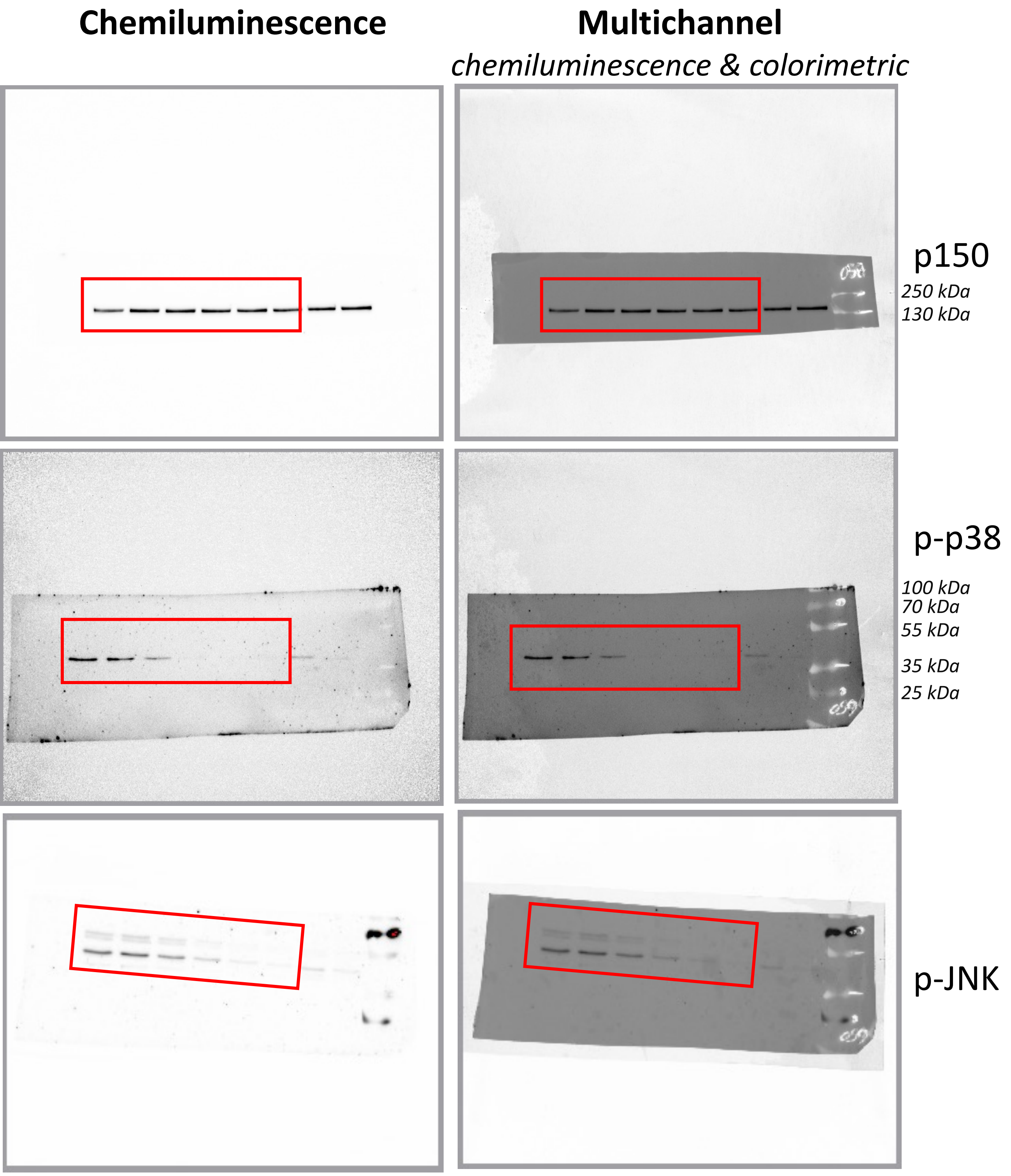

From the **left (the figure is mirrored in the manuscript!)**:

- **Lane 1:** *U2OS cells* – 1000 µM DEA NONOate, 1h
- **Lane 2:** *U2OS cells* – 750 µM DEA NONOate, 1h
- **Lane 3:** *U2OS cells* – 500 µM DEA NONOate, 1h
- **Lane 4:** *U2OS cells* – 250 µM DEA NONOate, 1h
- **Lane 5:** *U2OS cells* – 100 µM DEA NONOate, 1h
- **Lane 6**: *U2OS cells* – 1% DMSO, 1h
- **Lane 7**: *U2OS cells* – H_2O_2, 5 mM, 1h
- **Lane 8**: *U2OS cells* – Untreteated
- **Lane 9**:Ladder

Marked by the red square is the lanes included in the figure

Ryder et al, Figure 1b

Chemiluminescence

Multichannel
chemiluminescence & colorimetric

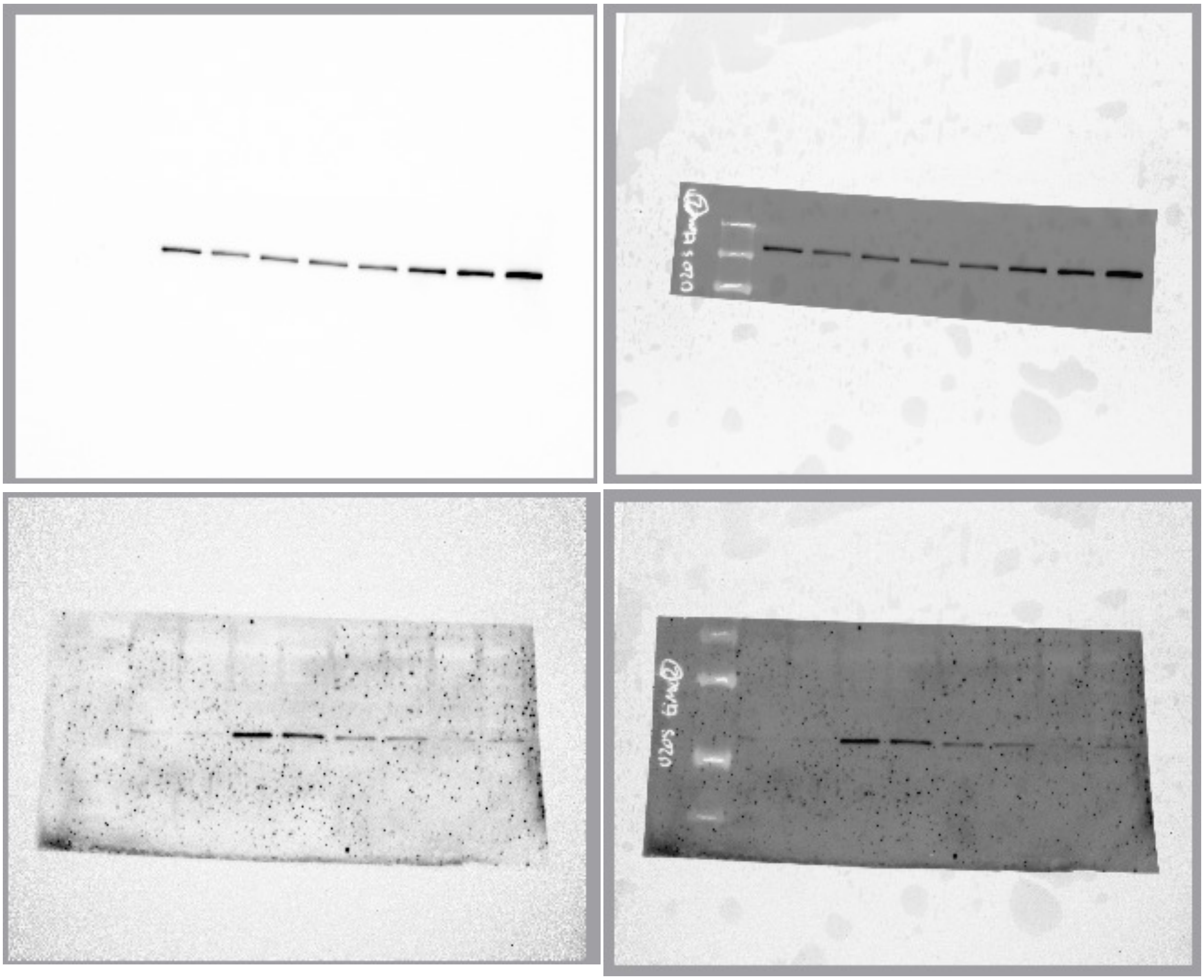

p150

250 kDa
130 kDa
100 kDa

p-p38

70 kDa
55 kDa
35 kDa
25 kDa

From the **left** :

- **Lane 1:** Ladder
- **Lane 2:** *U2OS cells* – Untreated
- **Lane 3:** *U2OS cells* – 750 **µ**M DEA NONOate, 15 min
- **Lane 4:** *U2OS cells* – 750 **µ**M DEA NONOate, 30 min
- **Lane 5:** *U2OS cells* – 750 **µ**M DEA NONOate, 1h
- **Lane 6:** *U2OS cells* – 750 **µ**M DEA NONOate, 2h
- **Lane 7**: *U2OS cells* – 750 **µ**M DEA NONOate, 4h
- **Lane 8:** *U2OS cells* – 750 **µ**M DEA NONOate, 6h
- **Lane 9**: *U2OS cells* – 750 **µ**M DEA NONOate, 8h

Chemiluminescence

Multichannel

chemiluminescence & colorimetric

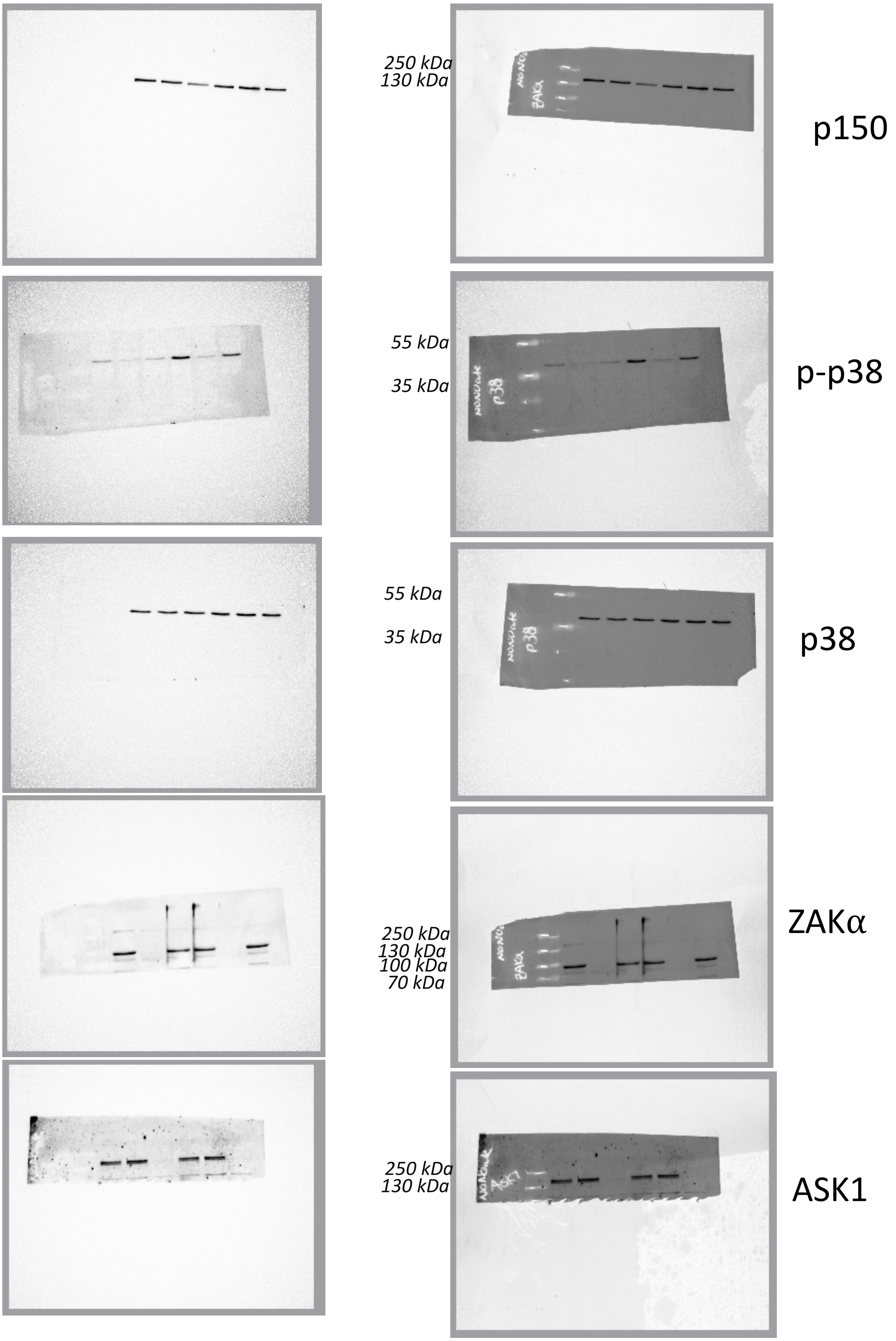

From the **left** :

- **Lane1: Ladder**
- **Lane 2:** *U2OS cells WT* – Untreated
- **Lane 3:** *U2OS cells ZAK KO* – Untreated
- **Lane 4:** *U2OS cells ASK1 KO* – Untreated
- **Lane 5:** *U2OS cells WT* – 750 **μ**M DEA NONOate, 1h
- **Lane 6:** *U2OS cells ZAK KO* – 750 **μ**M DEA NONOate, 1
- **Lane 7**: *U2OS cells* – 750 **μ**M DEA NONOate, 1h

Chemiluminescence

Multichannel

chemiluminescence & colorimetric

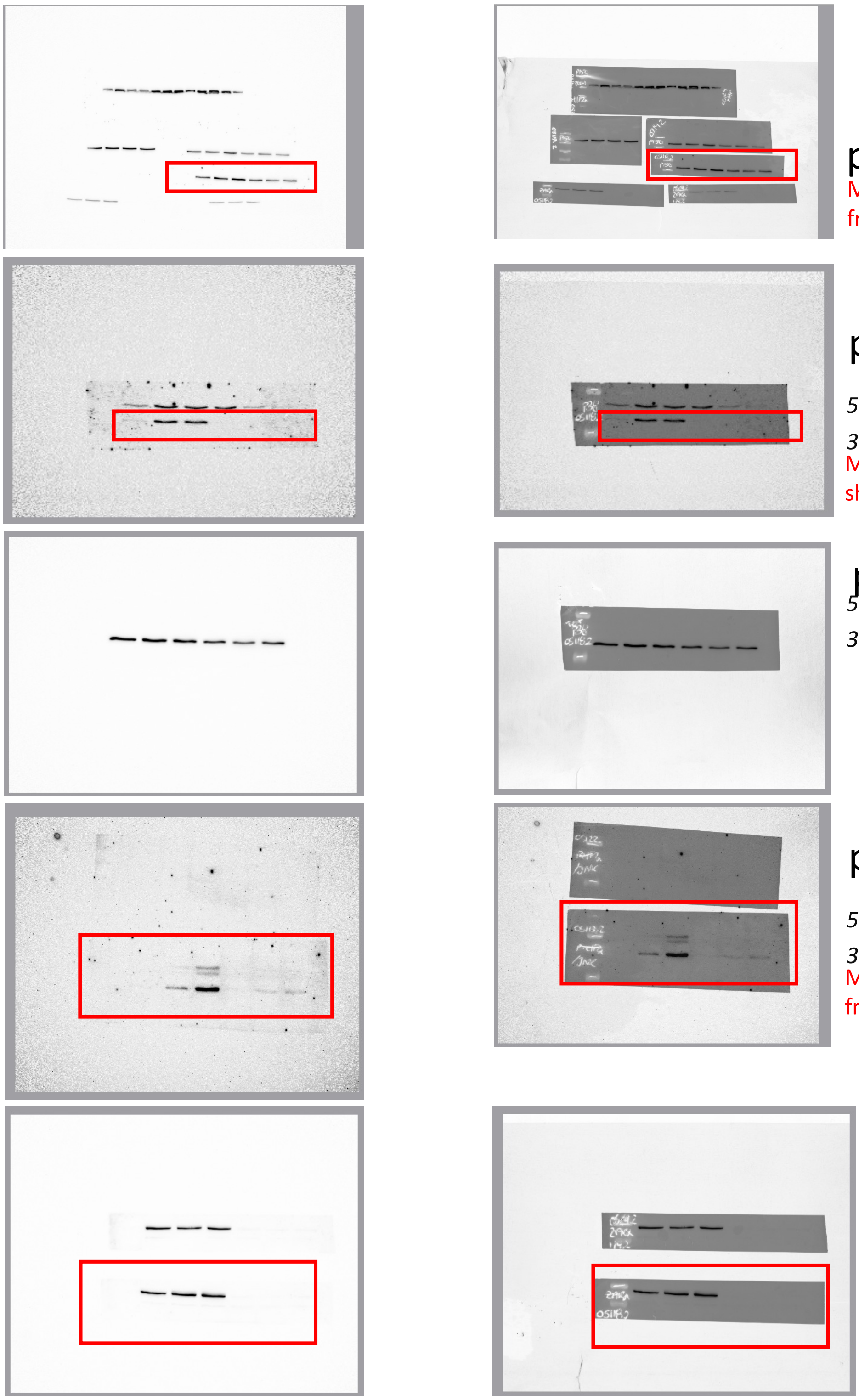

p150

Marked in red, membrane from this experiment

p-p38

55 kDa

35 kDa

Marked in red, the part shown in manuscript

p38

55 kDa

35 kDa

p-JNK

55 kDa

35 kDa

Marked in red, membrane from this experiment

ZAKα

100 kDa

Marked in red, membrane from this experiment

From the **left** :

- **Lane 1:** Ladder
- **Lane2:** *HAP1 cells WT* – Untreated
- **Lane 3:** *HAP1 cells WT* – 750 **µ**M DEA NONOate, 1h
- **Lane 4:** *HAP1 cells WT* – ASK1 2 **µ**M pre-treatmenrt + 750 **µ**M DEA NONOate, 1h
- **Lane 5:** *HAP1 cells ZAK KO* – Untreated
- **Lane 6:** *HAP1 cells ZAK KO* – 750 **µ**M DEA NONOate, 1h
- **Lane 7:** *HAP1 cells ZAK KO* – ASK1 2 **µ**M pre-treatmenrt + 750 **µ**M DEA NONOate, 1h

Ryder et al, Figure 1e

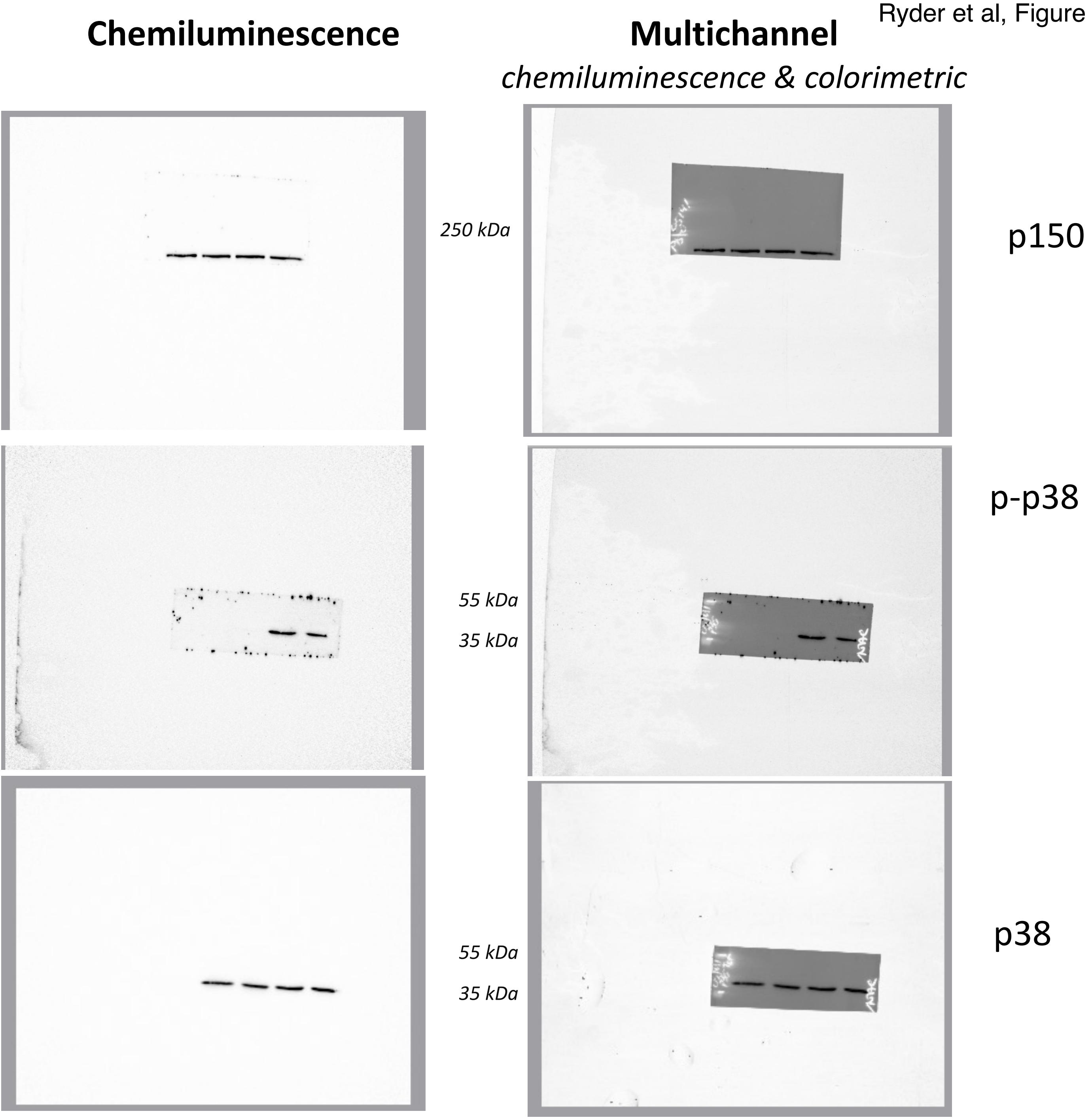

From the **left** :

- **Lane 1**: Ladder
- **Lane 2:***U2OS cells* – Untreated
- **Lane 3:** *U2OS cells* – 10 mM NAC, 1+1h
- **Lane 4:** *U2OS cells* – 750 **μ**M DEA NONOate, 1h
- **Lane 5:** *U2OS cells* – NAC 10 mM 1h pre-ytreatment + 750 **μ**M DEA NONOate, 1h

Chemiluminescence | **Multichannel**

chemiluminescence & colorimetric

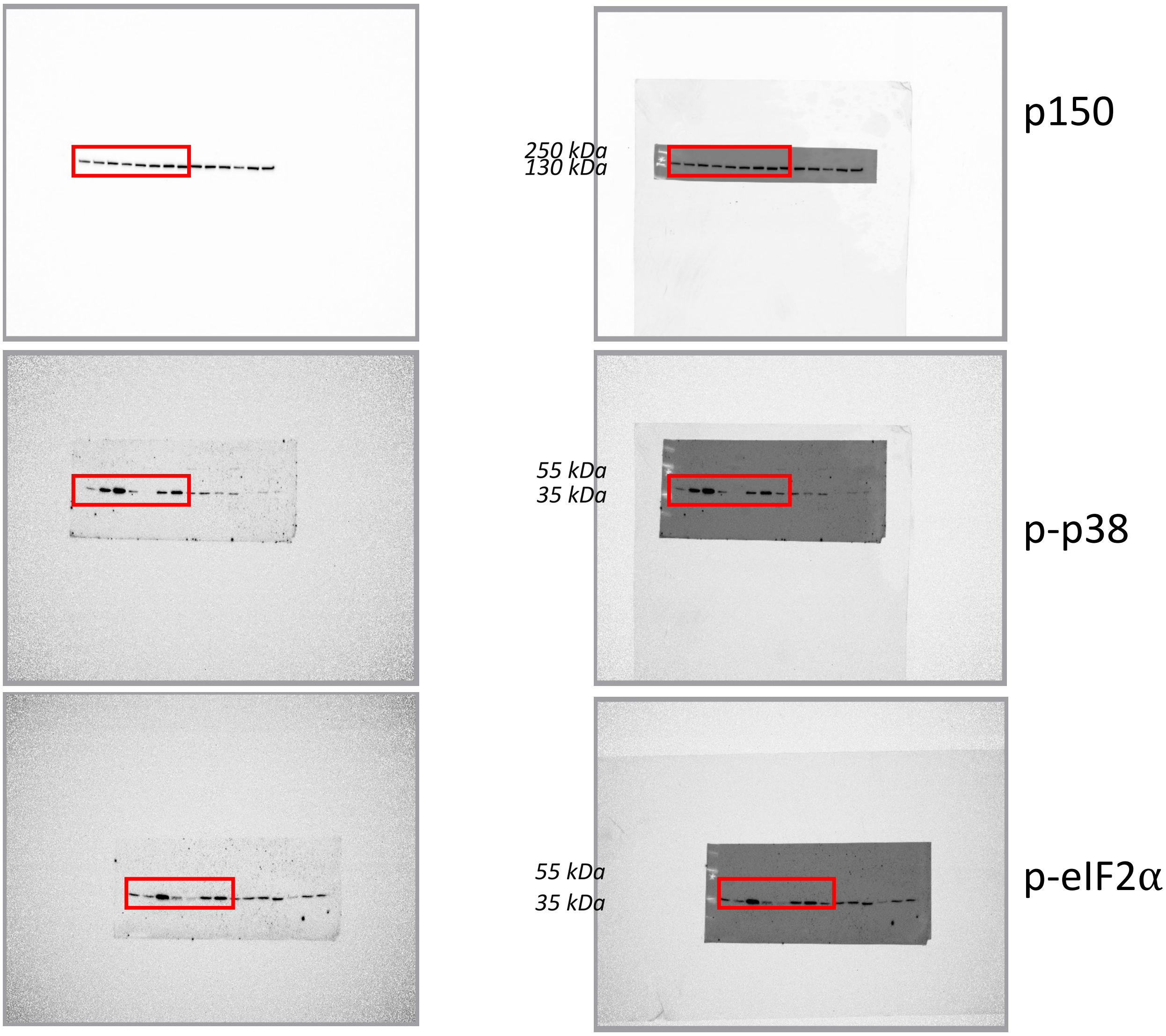

Marked by the red square is the lanes included in the figure

From the **left** :

- **Lane 1**: Ladder
- **Lane 2:***U2OS WT cells* – Untreated
- **Lane 3:** *U2OS WT cells* – 2mM GSNO, 1h
- **Lane 4:** *U2OS WT cells* – 2mM GSNO, 6h
- **Lane 5:** *U2OS WT cells* – 2mM GSNO, 24h
- **Lane 6:***U2OS ZAK KO cells* – Untreated
- **Lane 7:** *U2OS ZAK KO cells* – 2mM GSNO, 1h
- **Lane 8:** *U2OS ZAK KO cells* – 2mM GSNO, 6h
- **Lane 9:** *U2OS ZAK KO cells* – 2mM GSNO, 24h
- **Lane 10:** *U2OS WT cells* – 1% DMSO, 1h
- **Lane 11:** *U2OS WT cells* – 1% DMSO, 6h
- **Lane 12:** *U2OS WT cells* – 1% DMSO, 24h
- **Lane 13:** *U2OS ZAK KO cells* – 1% DMSO, 1h
- **Lane 14:** *U2OS ZAK KO cells* – 1% DMSO, 6h
- **Lane 15:** *U2OS WT cells* – 1% DMSO, 24h

Chemiluminescence | **Multichannel** *chemiluminescence & colorimetric*

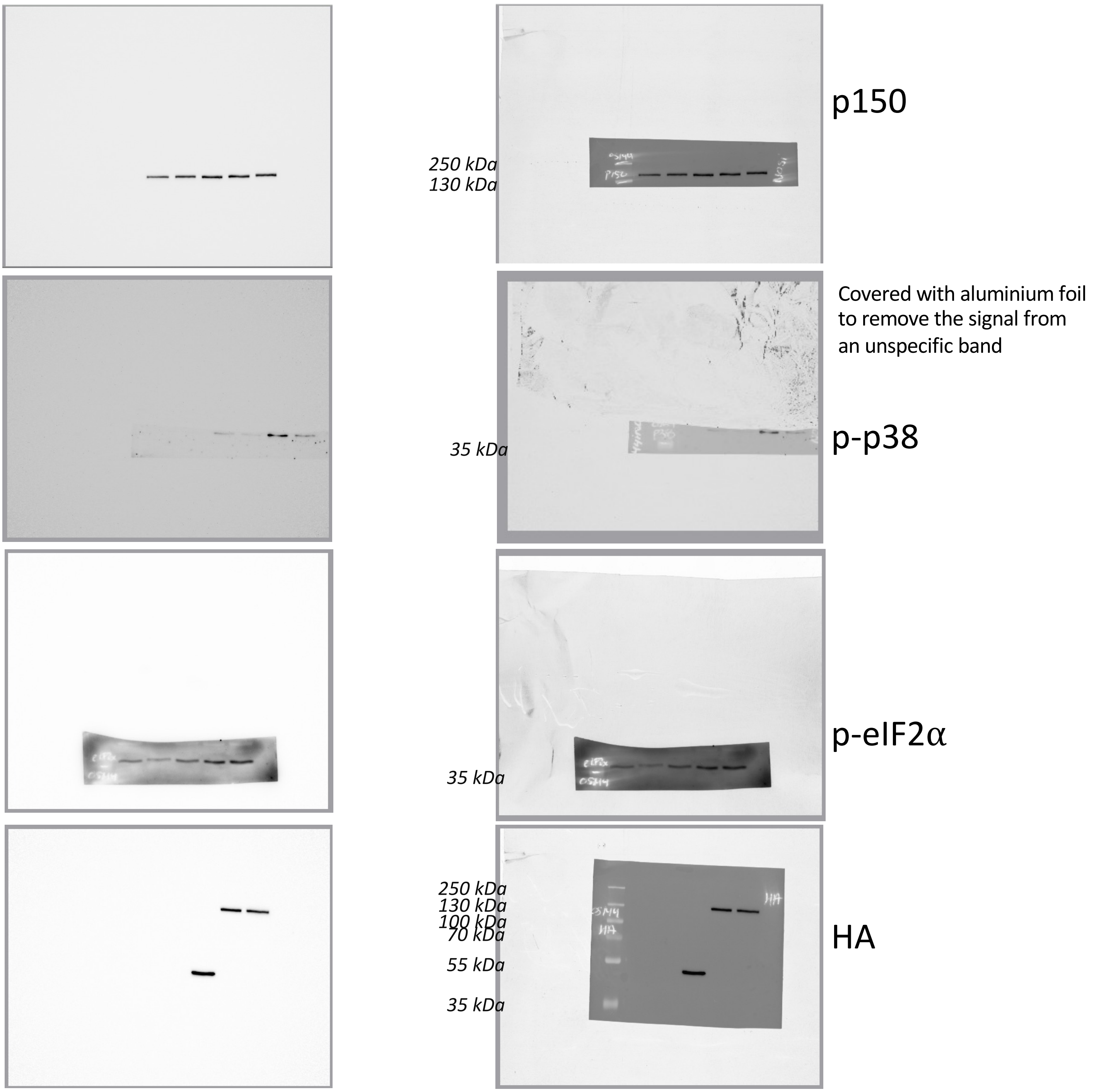

From the **left** :

- **Lane 1**: Ladder
- **Lane 2:** *U2OS cells* – Untreated
- **Lane 3:** *U2OS cells* – Transfected with empty strep-HA-vector
- **Lane 4:** *U2OS cells* – Transfected with HA-control plasmid
- **Lane 5:** *U2OS cells* – Transfected with HA-iNOS plasmid
- **Lane 6:** *U2OS cells* – Transfected with HA-iNOS plasmid + treated with ZAK inhibitor

Ryder et al, Figure 1h

Chemiluminescence **Multichannel**

chemiluminescence & colorimetric

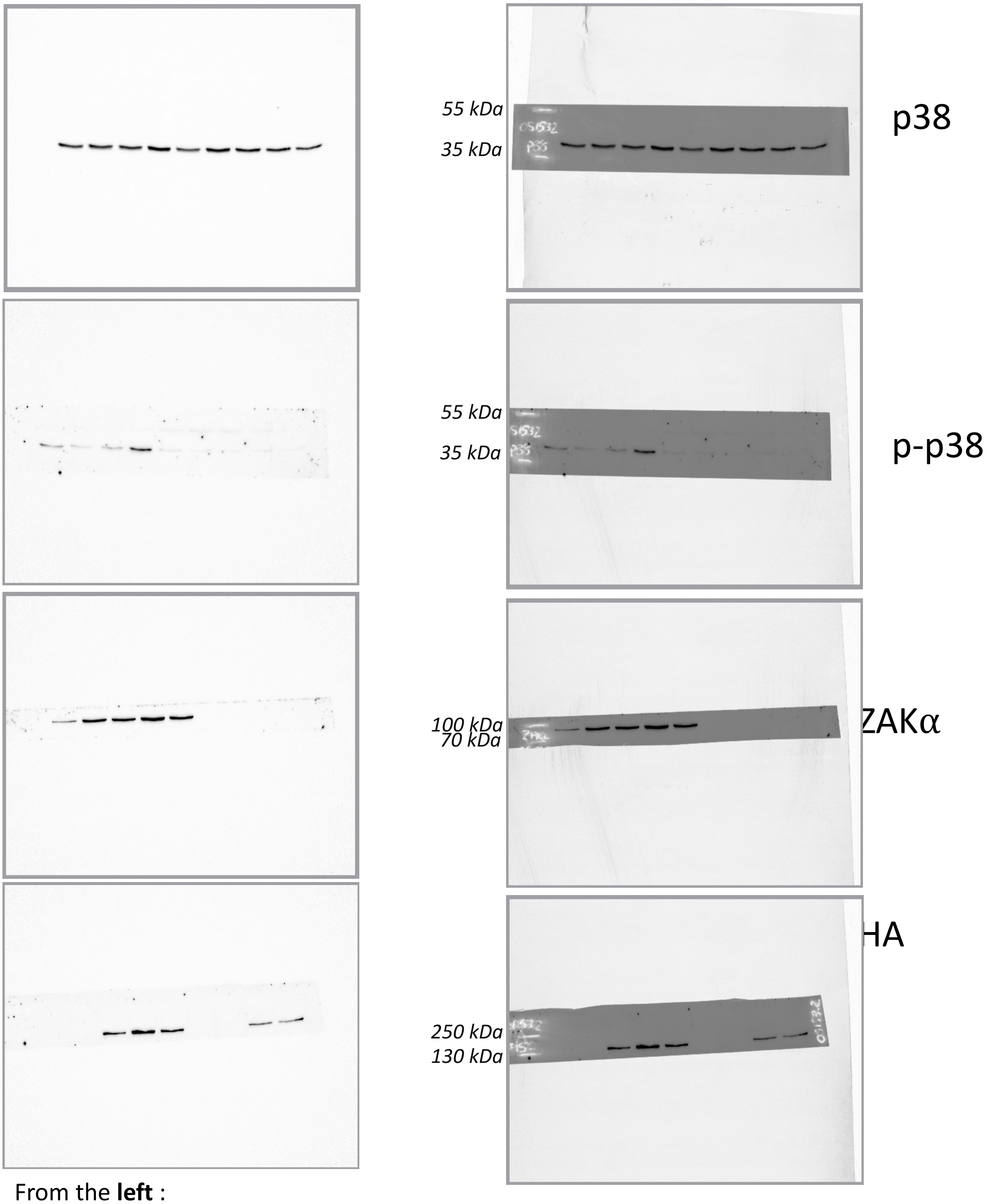

From the **left** :

- **Lane 1**: Ladder
- **Lane 2:** *U2OS WT cells* – Untreated
- **Lane 3:** *U2OS WT cells* – Transfected with empty strep-HA-vector
- **Lane 4:** *U2OS WT cells* – Transfected with HA-iNOS plasmid
- **Lane 5:** *U2OS WT cells* – Transfected with HA-iNOS plasmid + treated with 10 mM L-Arg, 20h
- **Lane 6:** *U2OS WT cells* – Transfected with HA-iNOS plasmid + treated with 10 mM L-Arg, 20h + treated with ZAK inhibitor, 1h
- **Lane 7:** *U2OS ZAK KO cells* – Untreated
- **Lane 8:** *U2OS ZAK KO cells* – Transfected with empty strep-HA-vector
- **Lane 9:** *U2OS ZAK KO cells* – Transfected with HA-iNOS plasmid
- **Lane 10:** *U2OS ZAK KO cells* – Transfected with HA-iNOS plasmid + treated with 10 mM L-Arg, 20h

Ryder et al, Figure 2b

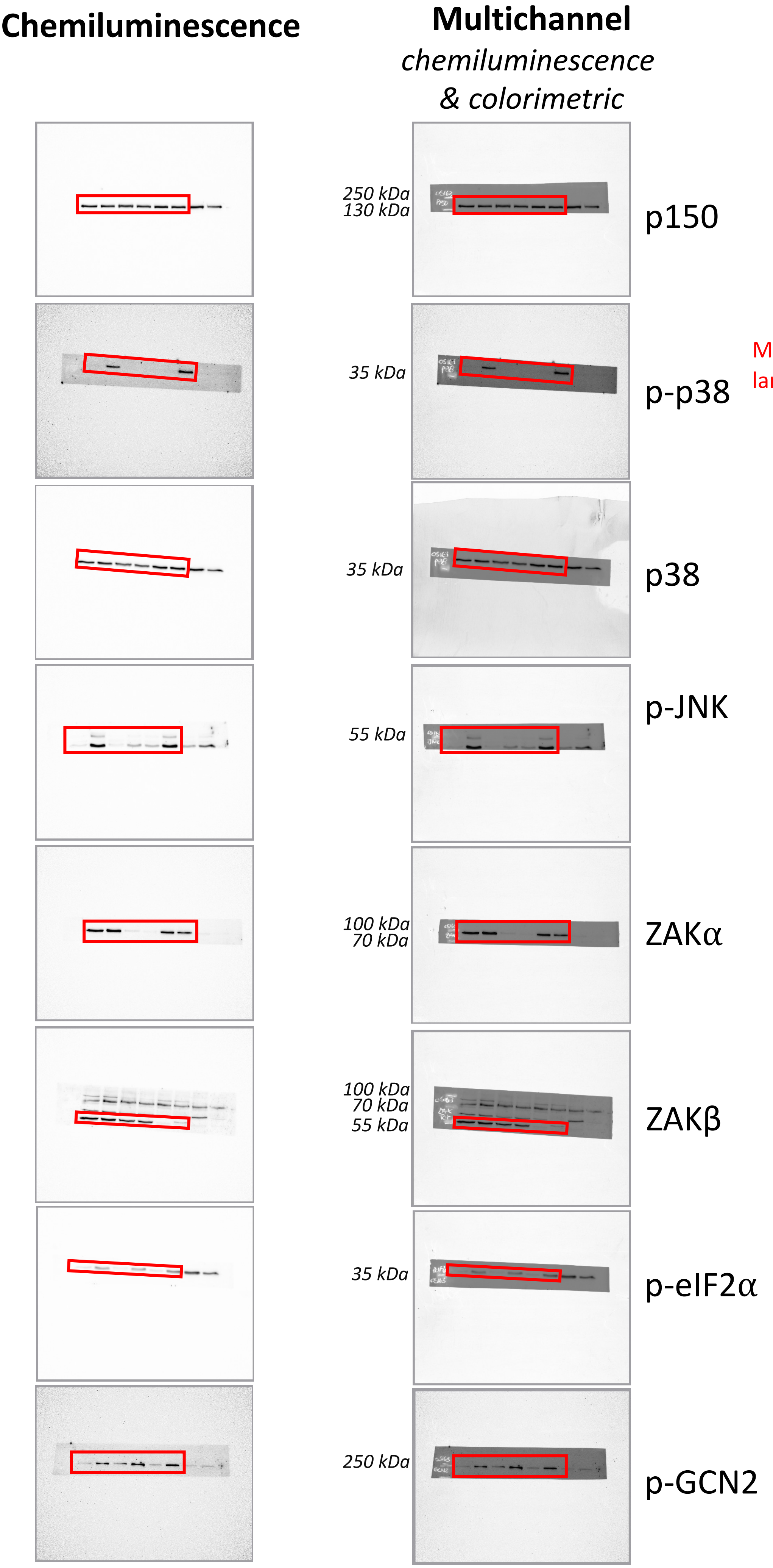

Marked by the red square is the lanes included in the figure

From the **left** :

- **Lane 1**: Ladder
- **Lane 2:** *U2OS cells* – Mock treated (reagents, but no siRNA)
- **Lane 3:** *U2OS cells* – Mock treated + 750 **µ**M DEA NONOate, 1h
- **Lane 4:** *U2OS cells* – siZAKα
- **Lane 5:** *U2OS cells* – siZAKα + 750 **µ**M DEA NONOate, 1h
- **Lane 6:** *U2OS cells* – siZAKβ
- **Lane 7:** *U2OS cells* – siZAKβ + 750 **µ**M DEA NONOate, 1h
- **Lane 8:** *U2OS cells* – siZAKαβ
- **Lane 9:** *U2OS cells* – siZAKαβ + 750 **µ**M DEA NONOate, 1h

Ryder et al, Figure 2c

Chemiluminescence

Multichannel
chemiluminescence
& colorimetric

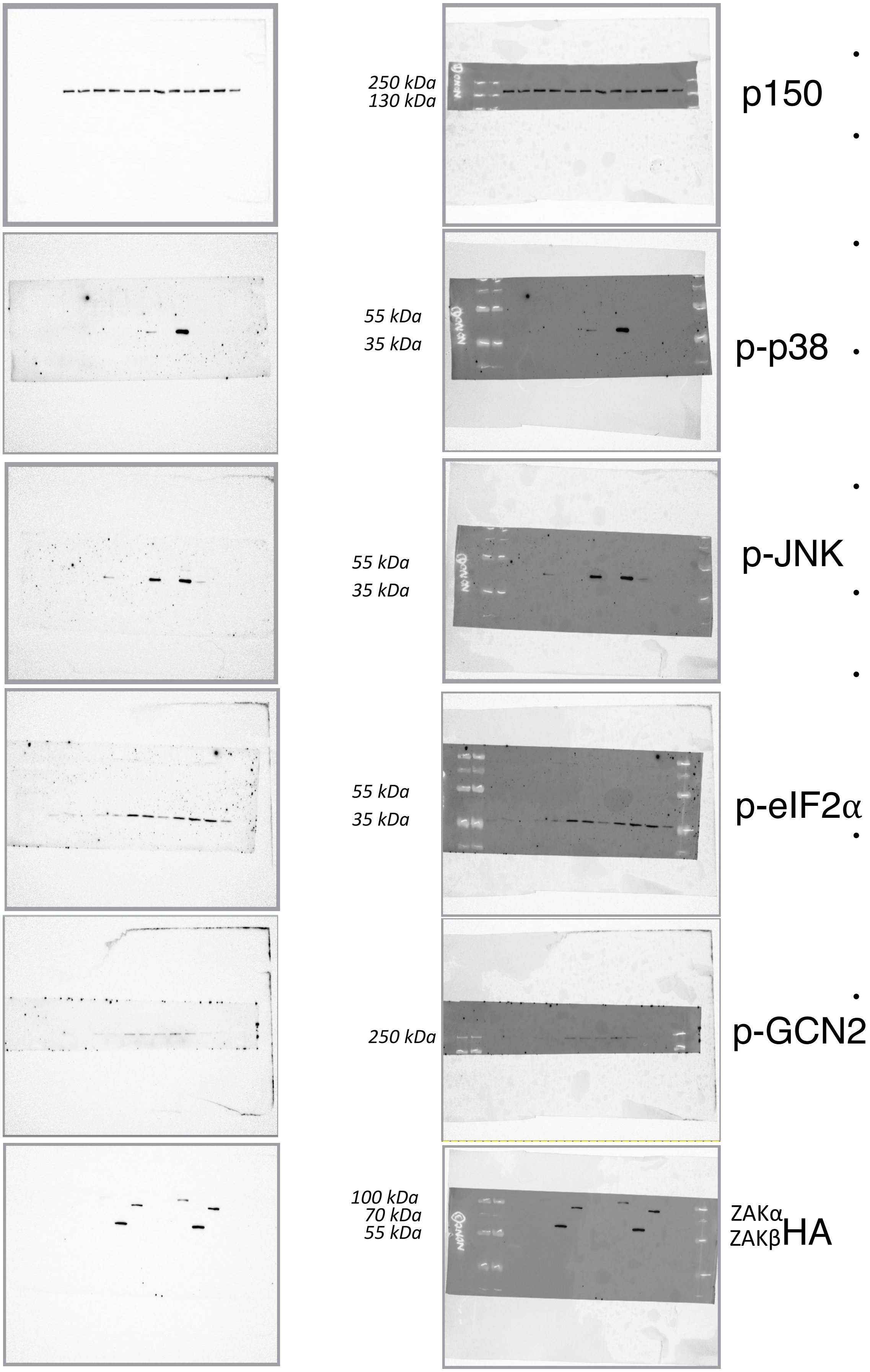

From the **left** :

- **Lane 1**: Ladder
- **Lane 2:** *U2OS cells* – Mock
- **Lane 3:** *U2OS ZAK KO cells* – Mock
- **Lane 4:** *U2OS ZAK KO inducible for* ZAKα – DOX-induced O/N
- **Lane 5:** *U2OS ZAK KO inducible for* ZAK β – DOX-induced O/N
- **Lane 6:** *U2OS ZAK KO inducible for* ZAK ΔαΔΔ – DOX-induced O/N
- **Lane 7:** *U2OS cells* + 750 **μ**M DEA NONOate, 1h
- **Lane 8:** *U2OS ZAK KO cells* – Mock
- **Lane 9:** *U2OS ZAK KO inducible for* ZAKα – DOX-induced O/N + 750 **μ**M DEA NONOate, 1h
- **Lane 10:** *U2OS ZAK KO inducible for* ZAK β – DOX-induced O/N + 750 **μ**M DEA NONOate, 1h
- **Lane 11:** *U2OS ZAK KO inducible for* ZAK ΔαΔΔ – DOX-induced O/N + 750 **μ**M DEA NONOate, 1h

Chemiluminescence

Multichannel
chemiluminescence & colorimetric

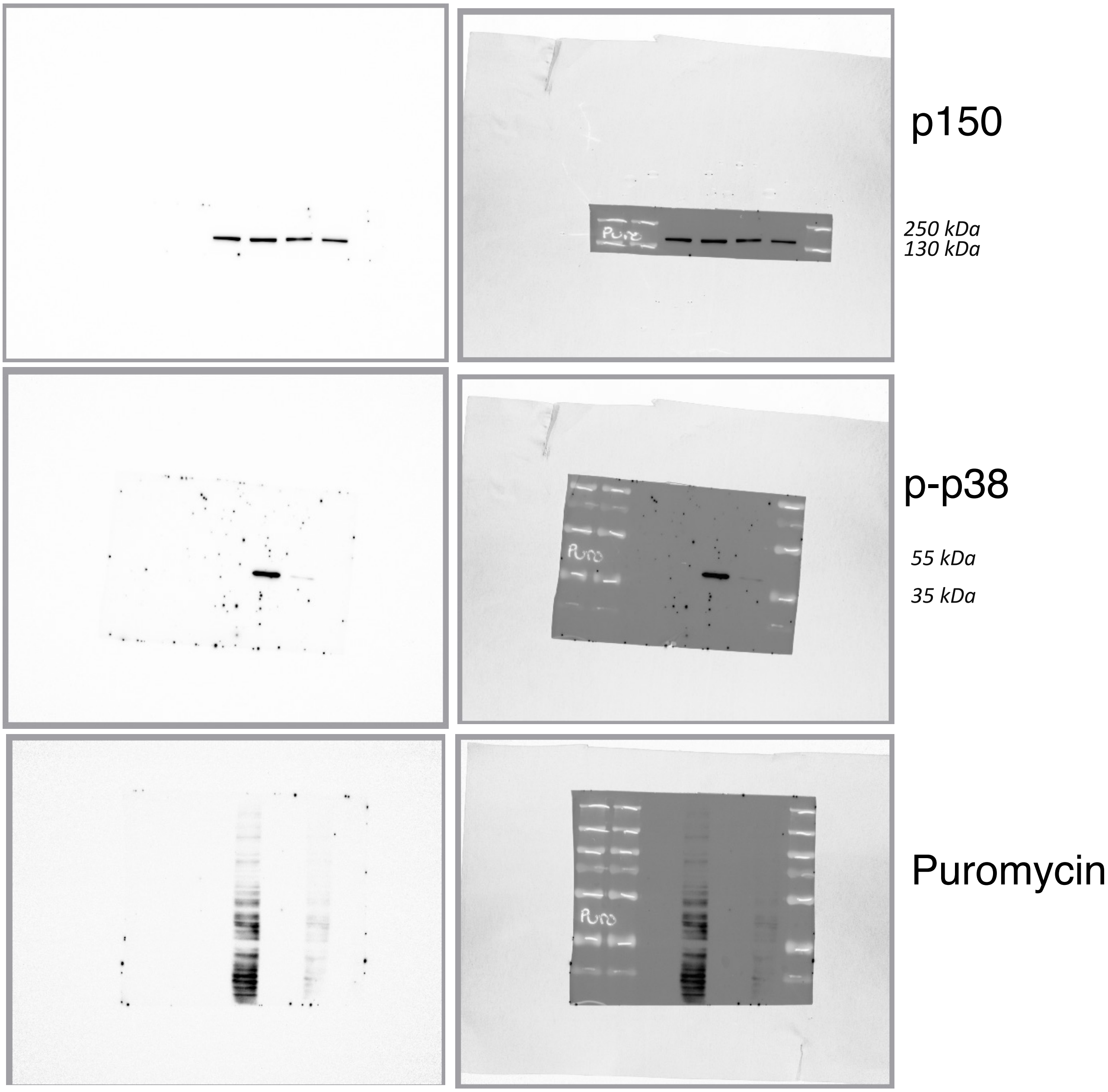

From the **left** :

- **Lane 1**: Ladder
- **Lane 2:** *U2OS cells* – Untreated
- **Lane 3:** *U2OS cells* – Puromycin 10 min
- **Lane 4:** *U2OS cells* – Anisomycin 1h + Puromycin 10 min
- **Lane 5:** *U2OS cells* – 750 **µ**M DEA NONOate 1h + Puromycin 10 min

Ryder et al, Figure 3b

Chemiluminescence

Multichannel
chemiluminescence
& colorimetric

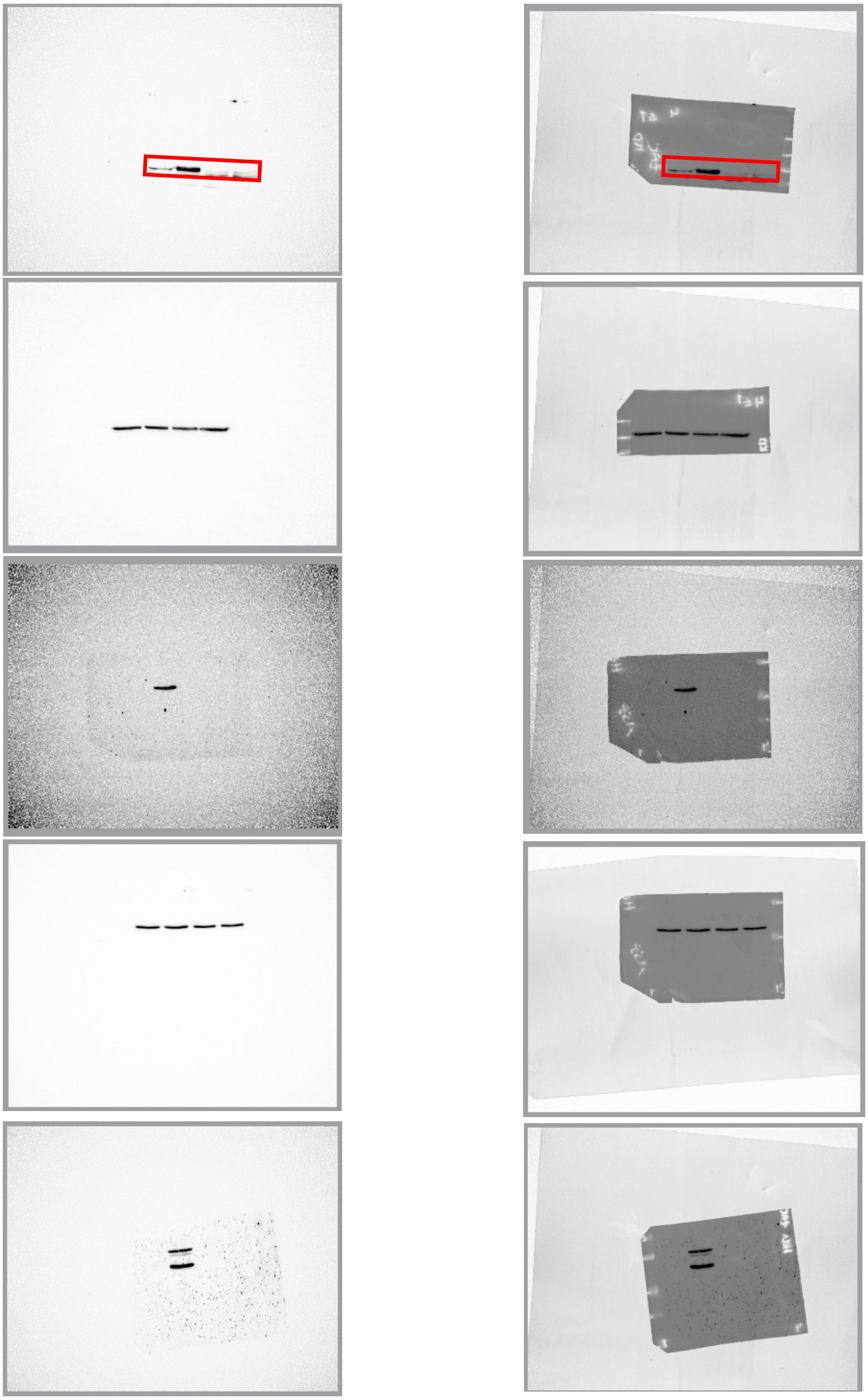

Marked by the red square is the lanes included in the figure

ZAKα
100 kDa
70 kDa

p150
250 kDa
130 kDa

p-p38
55 kDa
35 kDa

55 kDa
35 kDa
p38

p-JNK

From the **left** :

- **Lane 1**: *HeLa cells* Mock
- **Lane 2:** *HeLa cells* 750 **μ**M DEA NONOate 1h
- **Lane 3:** *HeLa cells* siZAKαβ

Lane 4: *HeLa cells* siZAKαβ + 750 **μ**M DEA NONOate 1h

Loading of gel – same samples were loaded twice onto the same gel divided by a ladder)

- **Lane 1**: *HeLa cells* – Mock
- **Lane 2:** *HeLa cells* – 750 **μ**M DEA NONOate 1h
- **Lane 3:** *HeLa cells* – siZAKαβ

Lane 4: *HeLa cells* – siZAKαβ + 750 **μ**M DEA NONOate 1h

} Blotted for ZAKα, p-p38, p38

Lane 5: *Ladder (membrane cut in the middle of this lane prior to antibody incubations)*

- **Lane 6/1**: *HeLa cells* – Mock
- **Lane 7/2:** *HeLa cells* – 750 **μ**M DEA NONOate 1h
- **Lane 8/3:** *HeLa cells* – siZAKαβ

Lane 9/4: *HeLa cells* – siZAKαβ + 750 **μ**M DEA NONOate 1h

} Blotted for p150, p-JNK

Ryder et al, Figure 4a

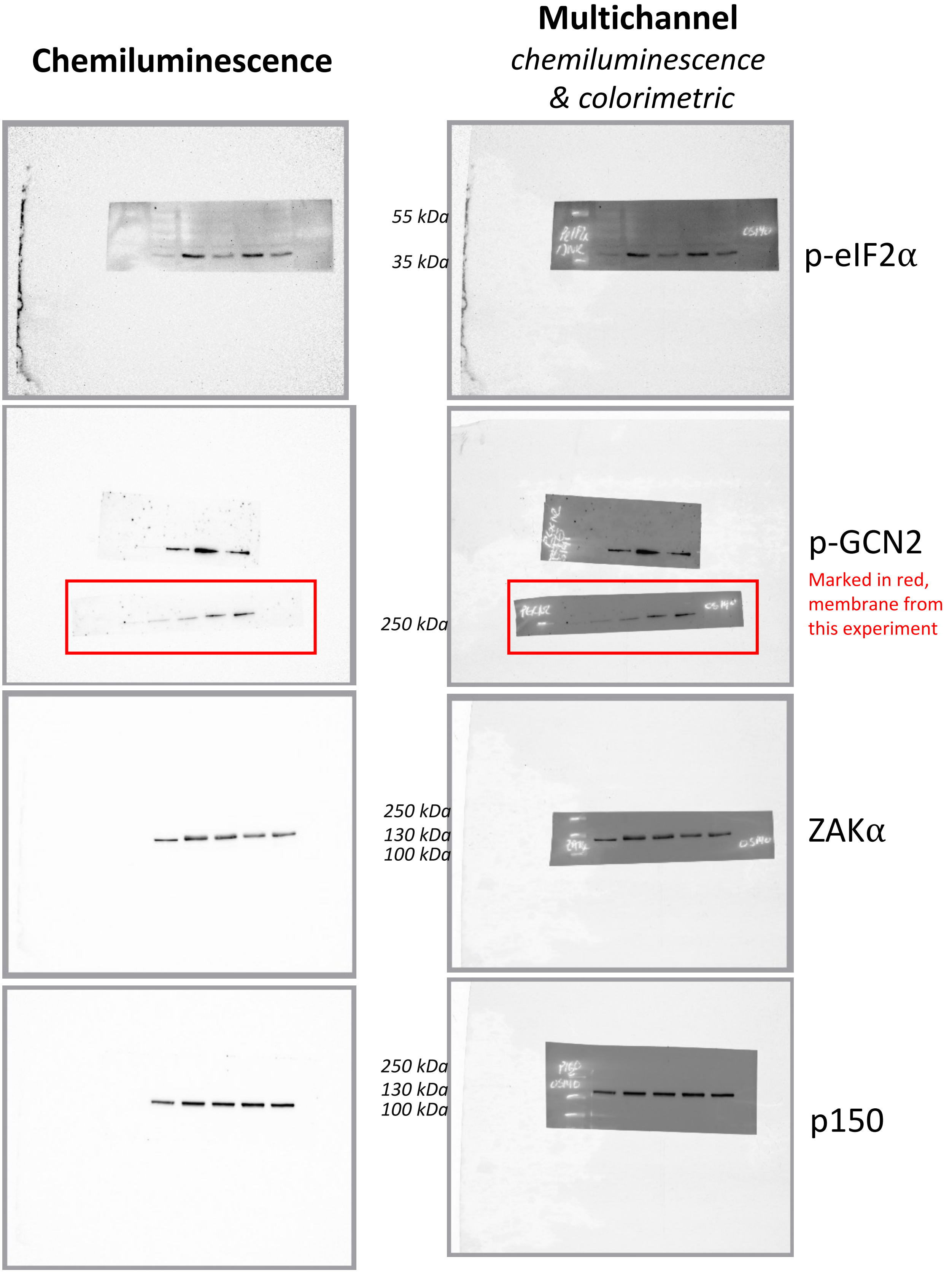

From the **left** :

- **Lane 1**: Ladder
- **Lane 2:** *U2OS cells* – Untreated
- **Lane 3:** *U2OS cells* – 750 **µ**M DEA NONOate 1h
- **Lane 4:** *U2OS cells* – GCN2 inhibitor 2 **µ**M, 30 min pre-treatment + 750 **µ**M DEA NONOate 1h
- **Lane 5:** *U2OS cells* – PERK inhibitor 2 **µ**M, 30 min pre-treatment + 750 **µ**M DEA NONOate 1h
- **Lane 6:** *U2OS cells* – GCN2 + PERK inhibitors 2 **µ**M , 30 min pre-treatment + 750 **µ**M DEA NONOate 1h

Multichannel
chemiluminescence & colorimetric

Chemiluminescence

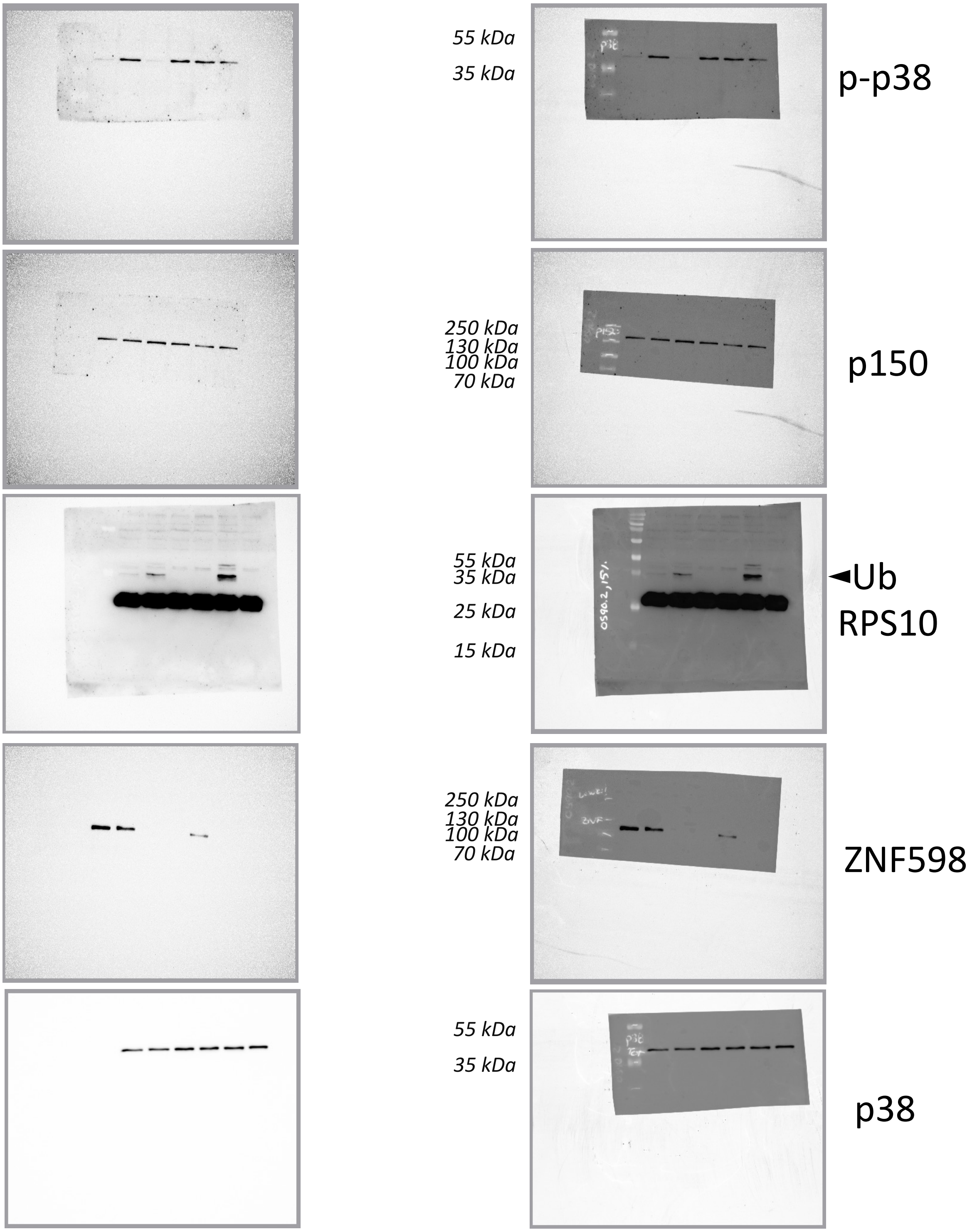

From the **left** :

- **Lane 1**: Ladder
- **Lane 2:** *U2OS WT cells* – Untreated
- **Lane 3:** *U2OS WT cells* – 750 **µ**M DEA NONOate 1h
- **Lane 4:** *U2OS ZNF598 KO cells* – Untreated
- **Lane 5:** *U2OS ZNF598 KO cells* – 750 **µ**M DEA NONOate 1h
- **Lane 6:** *U2OS WT cells* – Emetine, 1,8 µM, 15 min
- **Lane 7:** *U2OS ZNF598 KO cells* – Emetine, 1,8 µM, 15 min

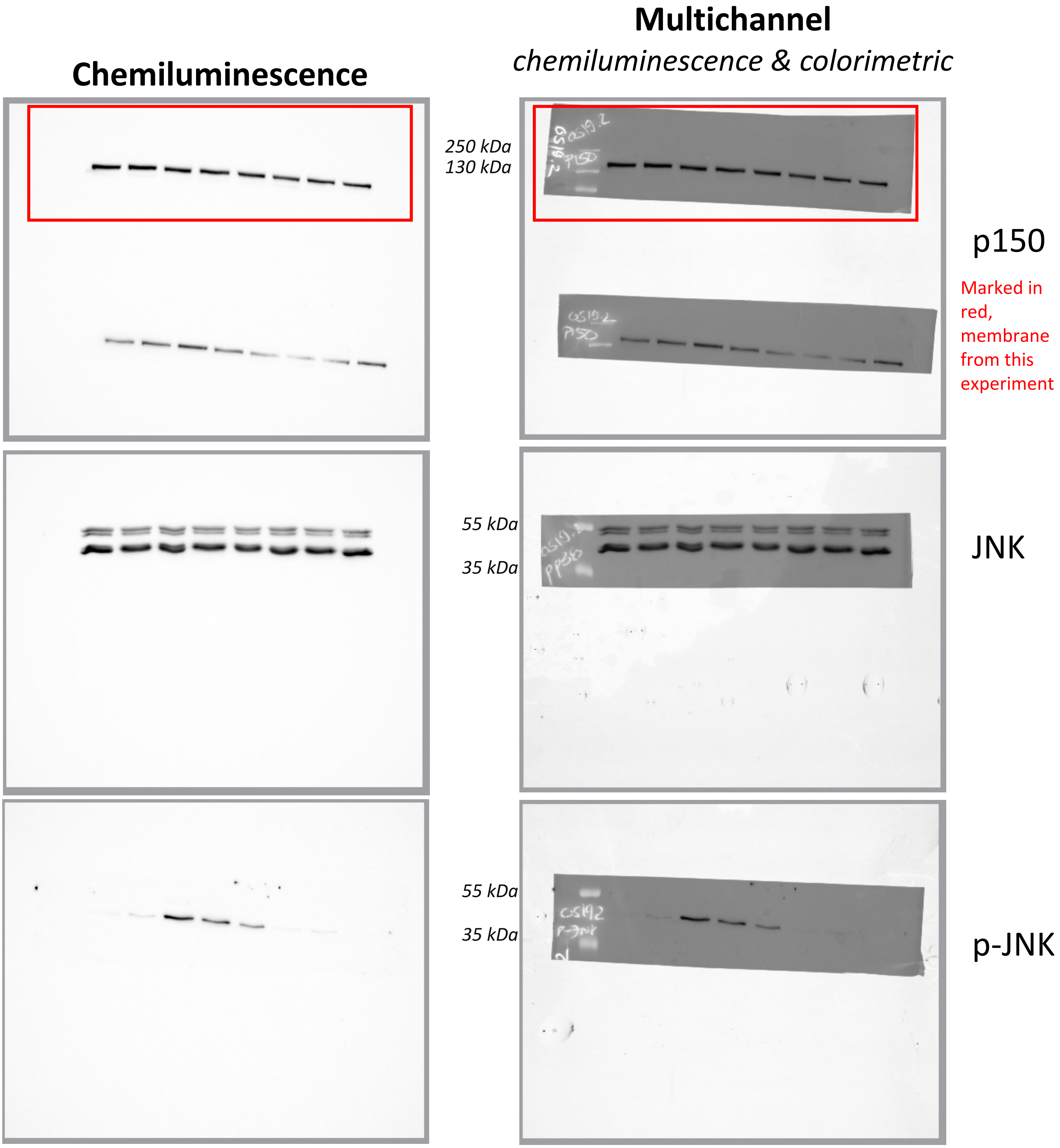

From the **left** :

- **Lane 1:** Ladder
- **Lane 2:** *U2OS cells* – Untreated
- **Lane 3:** *U2OS cells* – 750 µM DEA NONOate, 15 min
- **Lane 4:** *U2OS cells* – 750 µM DEA NONOate, 30 min
- **Lane 5:** *U2OS cells* – 750 µM DEA NONOate, 1h
- **Lane 6:** *U2OS cells* – 750 µM DEA NONOate, 2h
- **Lane 7**: *U2OS cells* – 750 µM DEA NONOate, 4h
- **Lane 8:** *U2OS cells* – 750 µM DEA NONOate, 6h
- **Lane 9**: *U2OS cells* – 750 µM DEA NONOate, 8h

Ryder et al, Figure S1b

Chemiluminescence

Multichannel
chemiluminescence & colorimetric

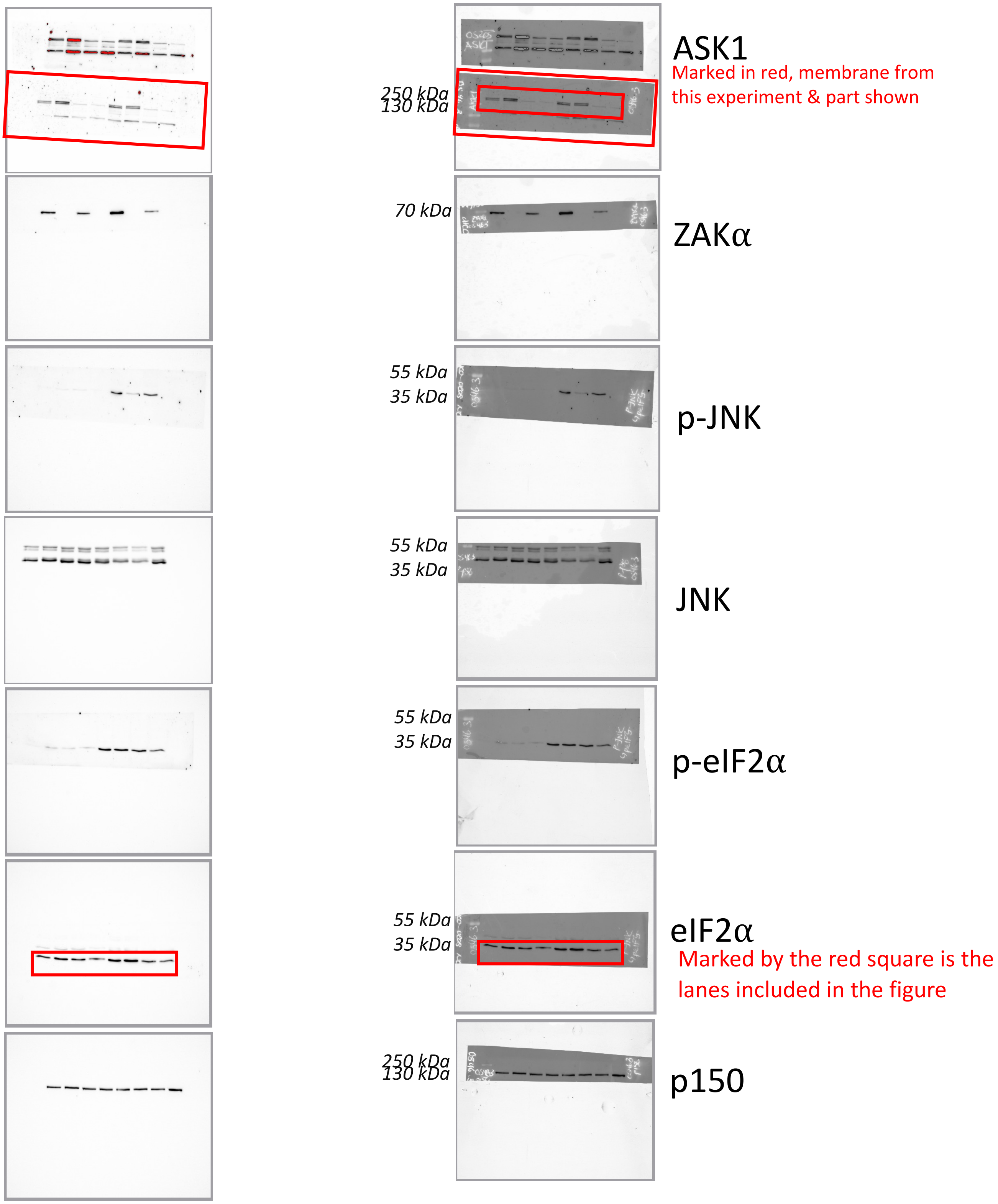

From the **left** :

- **Lane1: Ladder**
- **Lane 2:** *U2OS cells WT* – Untreated
- **Lane 3:** *U2OS cells ZAK KO* – Untreated
- **Lane 4:** *U2OS cells ASK1 KO* – Untreated
- **Lane 5:** *U2OS cells ZAK/ASK1 dKO* – Untreated
- **Lane 6:** *U2OS cells WT* – 750 **µ**M DEA NONOate, 1h
- **Lane 7:** *U2OS cells ZAK KO* – 750 **µ**M DEA NONOate, 1h
- **Lane 8**: *U2OS cells* – 750 **µ**M DEA NONOate, 1h
- **Lane 9:** *U2OS cells ZAK/ASK1 dKO* – 750 **µ**M DEA NONOate, 1h

Chemiluminescence

Multichannel

chemiluminescence & colorimetric

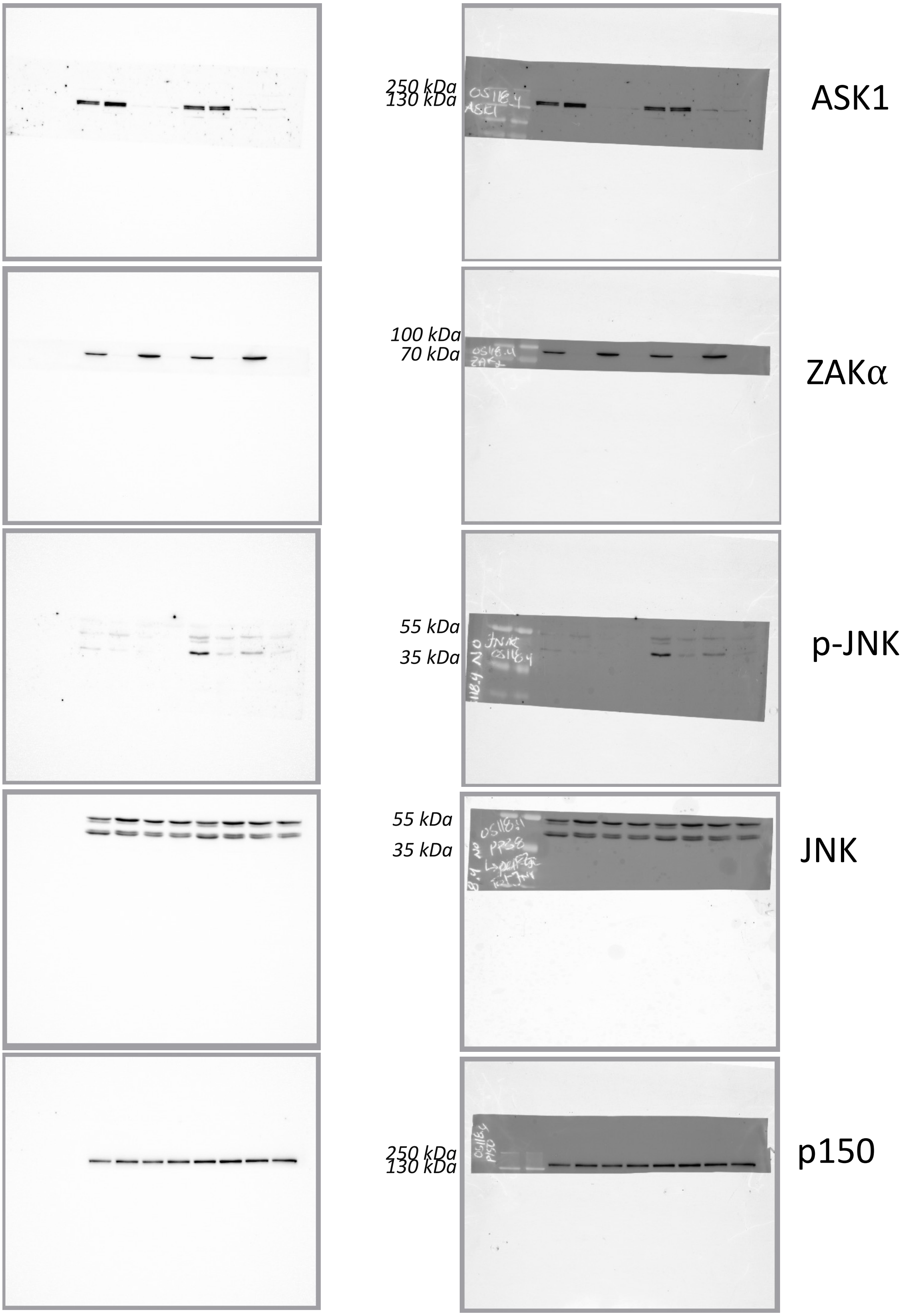

From the **left** :

- **Lane1:** Ladder
- **Lane2:** Ladder
- **Lane 3:** *HAP1 cells WT* – Untreated
- **Lane 4:** *HAP1 cells ZAK KO* – Untreated
- **Lane 5:** *HAP1 cells ASK1 KO* – Untreated
- **Lane 6:** *HAP1 cells ZAK/ASK1 dKO* – Untreated
- **Lane 7:** *HAP1 cells WT* – 750 **µ**M DEA NONOate, 1h
- **Lane 8:** *HAP1 cells ZAK KO* – 750 **µ**M DEA NONOate, 1h
- **Lane 9**: *HAP1 cells* – 750 **µ**M DEA NONOate, 1h
- **Lane 10:** *HAP1 cells ZAK/ASK1 dKO* – 750 **µ**M DEA NONOate, 1h

Chemiluminescence

Multichannel
chemiluminescence & colorimetric

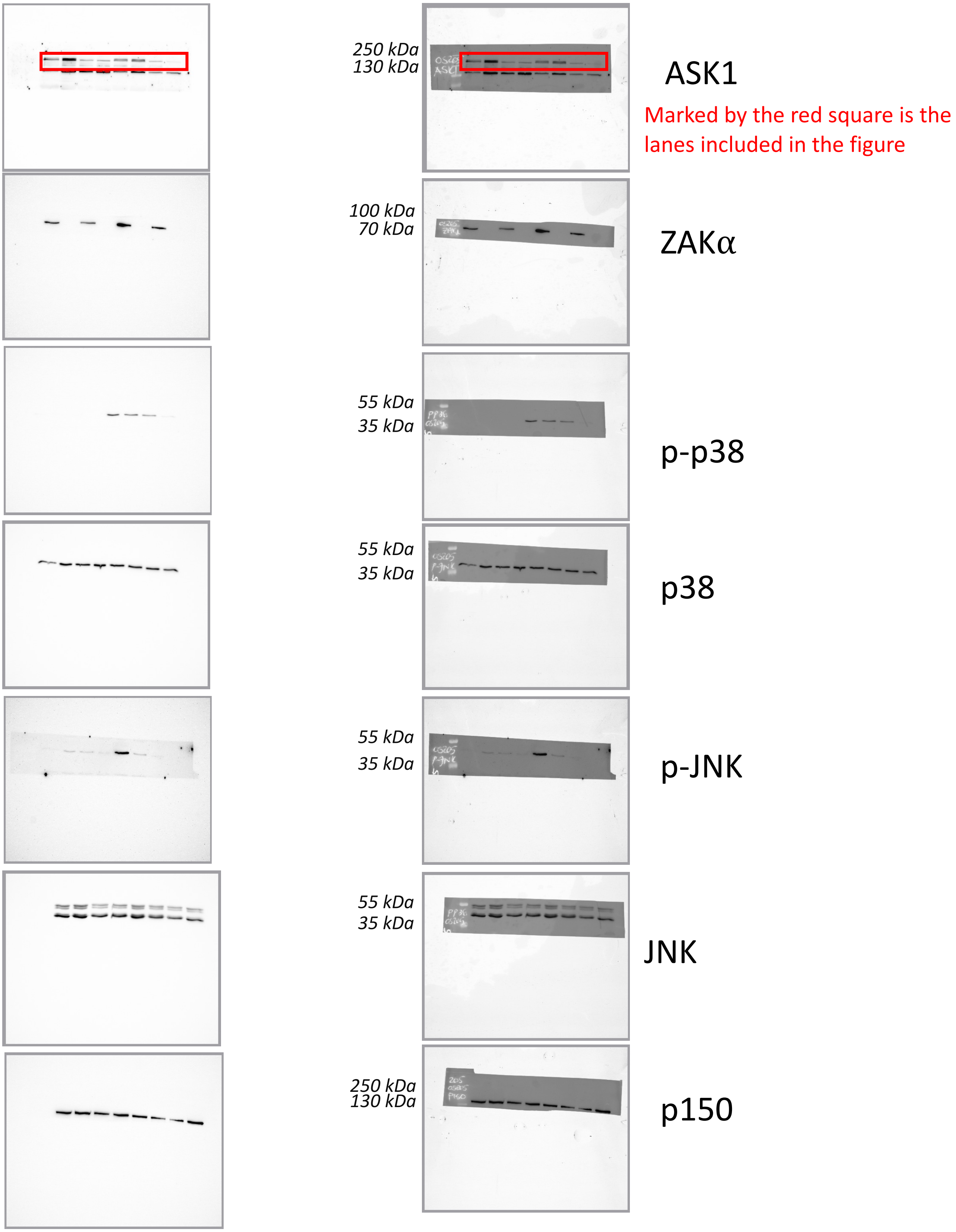

From the **left** :

- **Lane1: Ladder**
- **Lane 2:** *U2OS cells WT* – Untreated
- **Lane 3:** *U2OS cells ZAK KO* – Untreated
- **Lane 4:** *U2OS cells ASK1 KO* – Untreated
- **Lane 5:** *U2OS cells ZAK/ASK1 dKO* – Untreated
- **Lane 6:** *U2OS cells WT* – 2mM GSNO, 6h
- **Lane 7:** *U2OS cells ZAK KO* – 2mM GSNO, 6h
- **Lane 8:** *U2OS cells* – 2mM GSNO, 6h
- **Lane 9:** *U2OS cells ZAK/ASK1 dKO* – 2mM GSNO, 6h

Ryder et al, Figure S1e

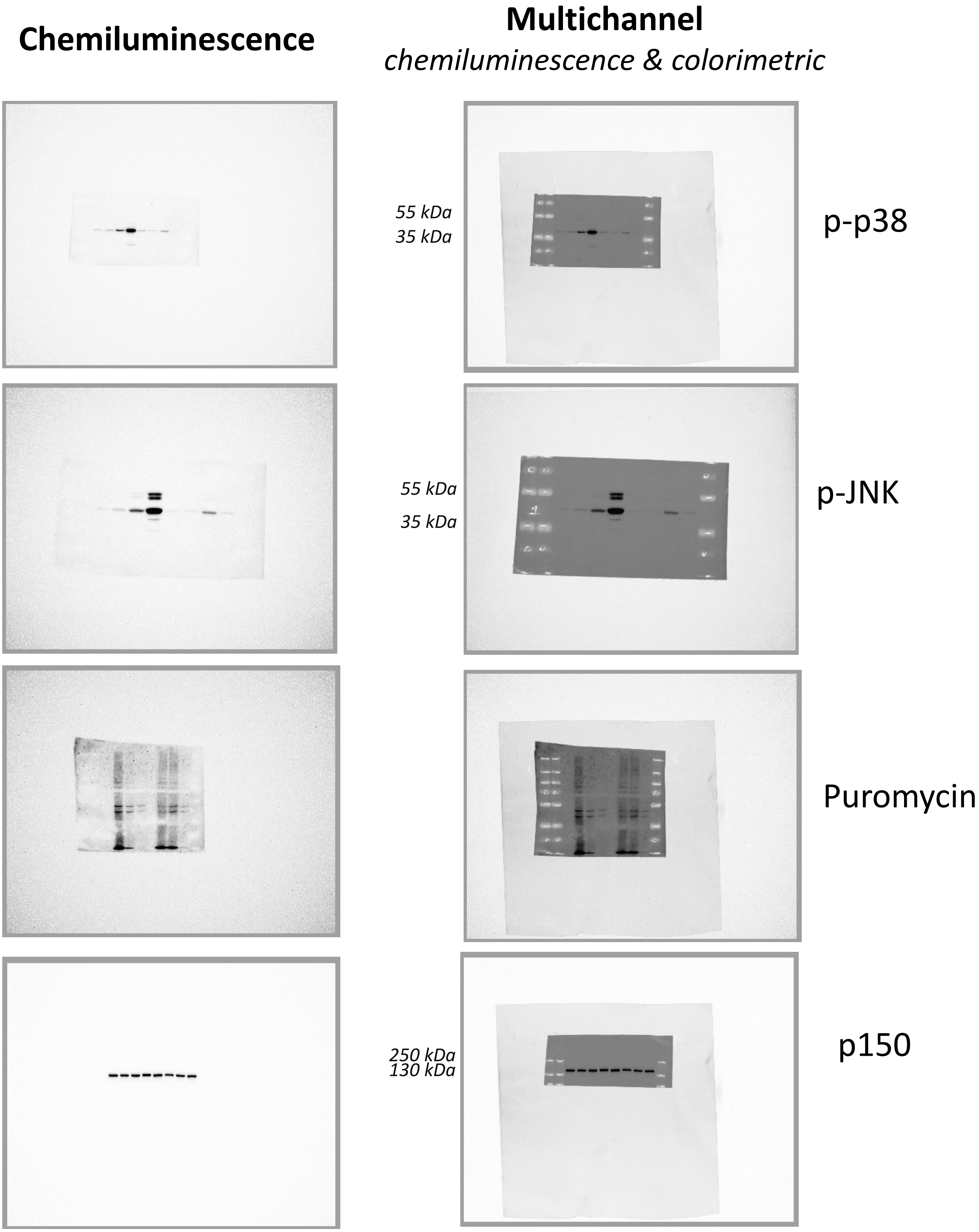

From the **left** :

- **Lane 1:** Ladder
- **Lane 2:** Ladder
- **Lane 3:** *U2OS WT cells* – Untreated
- **Lane 4:** *U2OS WT cells* – Puromycin 10 min
- **Lane 5:** *U2OS WT cells* – 2mM GSNO, 6h + Puromycin 10 min
- **Lane 6:** *U2OS ZAK KO cells* – Anisomycin, 1h + Puromycin 10 min
- **Lane 7:** *U2OS ZAK KO cells* – Untreated
- **Lane 8:** *U2OS ZAK KO cells* – Puromycin 10 min
- **Lane 9:** *U2OS ZAK KO cells* – 2mM GSNO, 6h + Puromycin 10 min
- **Lane 10:** *U2OS ZAK KO cells* – Anisomycin, 1h + Puromycin 10 min
- **Lane 11:** Ladder

Ryder et al, Figure S2a

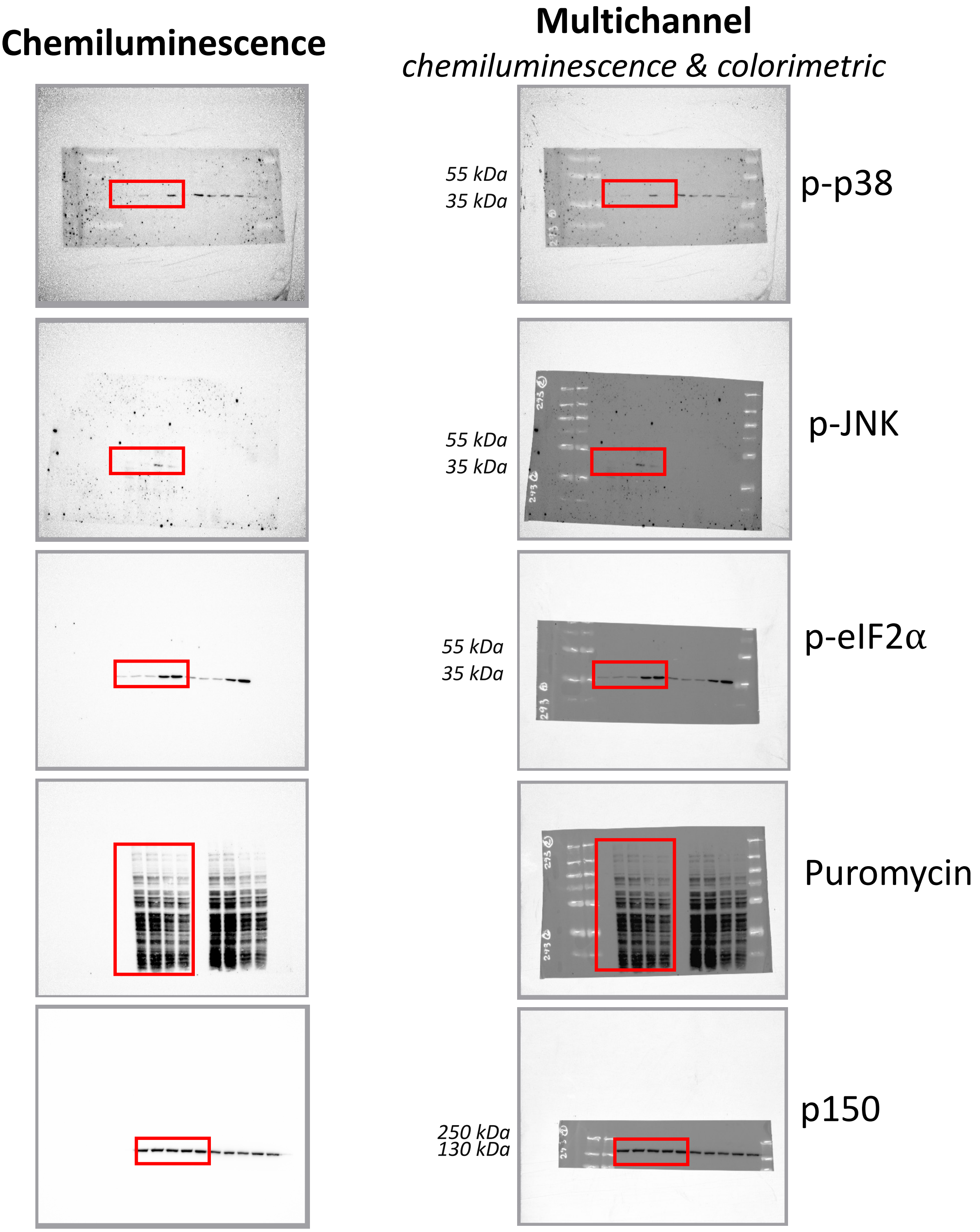

From the **left** :

- **Lane 1:** Ladder
- **Lane 2:** Ladder
- **Lane 3:** *HEK293 cells* – Untreated (in DMEM medium)
- **Lane 4:** *HEK293 cells* – Puromycin 10 min (in DMEM medium)
- **Lane 5:** *HEK293 cells* – 0,75% DMSO, 1h + Puromycin 10 min (in DMEM medium)
- **Lane 6:** *HEK293 cells* – 750 **μ**M DEA NONOate, 1h + Puromycin 10 min (in DMEM medium)
- **Lane 7:** *HEK293 cells* – ZAKi 2 **μ**M pre-treatmen+ 750 **μ**M DEA NONOate, 1h + Puromycin 10 min (in DMEM medium)
- **Lane 8:** *HEK293 cells* – Untreated (in RPMI medium, 1h)
- **Lane 9:** *HEK293 cells* – Puromycin 10 min in RPMI medium during treatment)
- **Lane 10:** *HEK293 cells* – 0,75% DMSO, 1h + Puromycin 10 min (in RPMI medium during treatment)
- **Lane 11:** *HEK293 cells* – 750 **μ**M DEA NONOate, 1h + Puromycin 10 min (in RPMI medium during treatment)
- **Lane 12:** *HEK293 cells* – ZAKi 2 **μ**M pre-treatmen+ 750 **μ**M DEA NONOate, 1h + Puromycin 10 min (in RPMI medium)
- **Lane 13:** Ladder

Marked by the red square is the lanes included in the figure

Chemiluminescence

Multichannel
chemiluminescence &

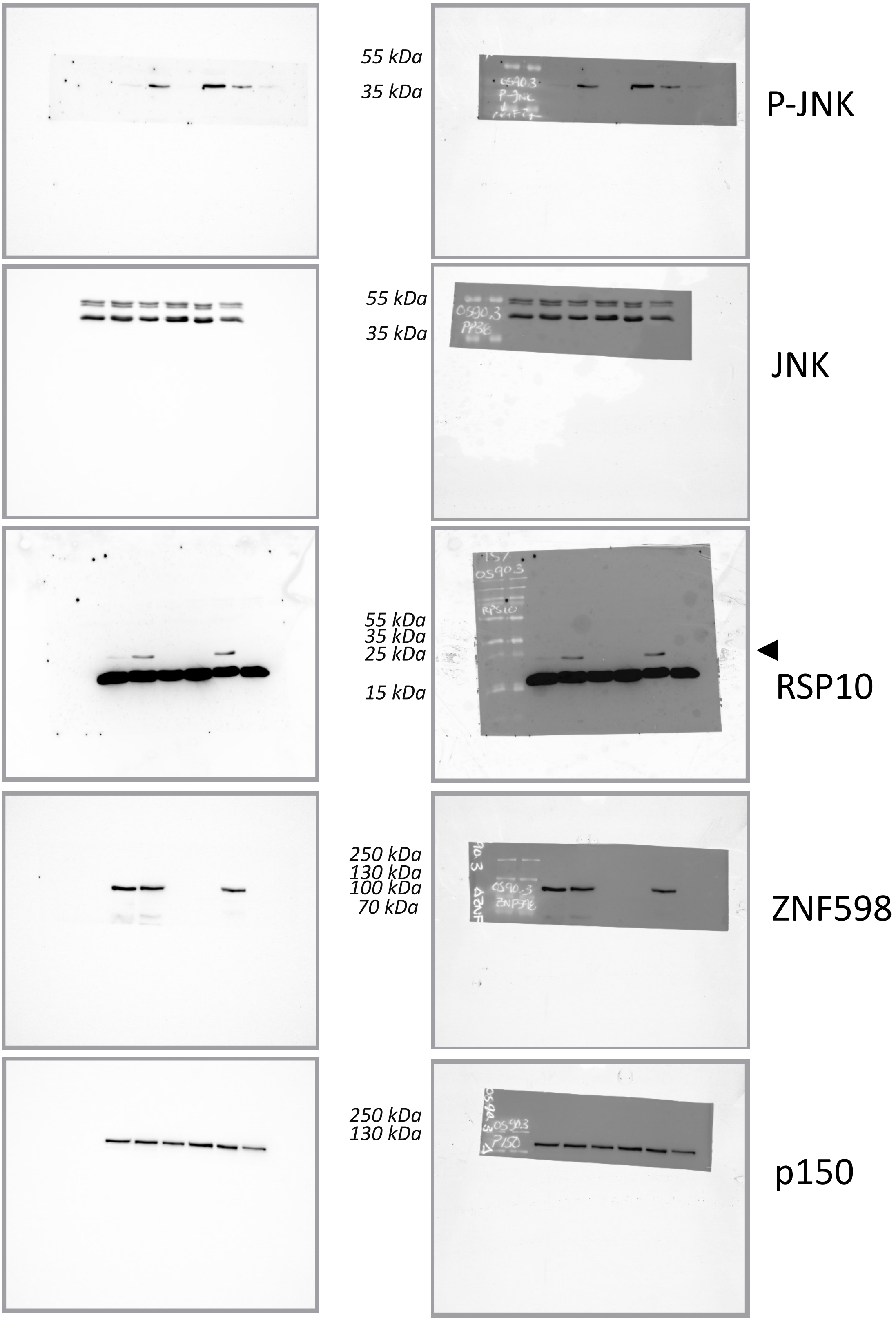

From the **left** :

- **Lane 1**: Ladder
- **Lane 2**: Ladder
- **Lane 3:** *U2OS WT cells* – Untreated
- **Lane 4:** *U2OS WT cells* – 750 **µ**M DEA NONOate 1h
- **Lane 5:** *U2OS ZNF598 KO cells* – Untreated
- **Lane 6:** *U2OS ZNF598 KO cells* – 750 **µ**M DEA NONOate 1h
- **Lane 7:** *U2OS WT cells* – Emetine, 1,8 µM, 15 min
- **Lane 8:** *U2OS ZNF598 KO cells* – Emetine, 1,8 µM, 15 min
